# FoxO3 restricts liver regeneration by suppressing the proliferation of hepatocytes

**DOI:** 10.1038/s41536-022-00227-6

**Published:** 2022-06-24

**Authors:** Chi-Qian Liang, Deng-Cheng Zhou, Wen-Tao Peng, Wu-Yun Chen, Hai-Yan Wu, Yi-Min Zhou, Wei-Li Gu, Kyu-Sang Park, Hui Zhao, Long-Quan Pi, Li Zheng, Shan-Shan Feng, Dong-Qing Cai, Xu-Feng Qi

**Affiliations:** 1 Key Laboratory of Regenerative Medicine of Ministry of Education, Department of Developmental & Regenerative Biology, College of Life Science and Technology, Jinan University, Guangzhou, 510632 China; 2 Department of Hepato-Pancreato-Biliary Surgery, Guangzhou First People’s Hospital, School of Medicine, South China University of Technology, Guangzhou, China; 3 Department of Physiology, Wonju College of Medicine, Yonsei University, Wonju, Gangwon 220-701 Korea; 4 Key Laboratory of Regenerative Medicine of Ministry of Education, School of Biomedical Sciences, Faculty of Medicine, The Chinese University of Hong Kong, Hong Kong, SAR China; 5 Department of Dermatology, Yanbian University Hospital, Yanji, Jilin, 133002 China; 6 School of Environmental Science and Engineering, Guangdong University of Technology, Guangzhou, 510006 China

**Keywords:** Experimental models of disease, Regenerative medicine

## Abstract

Upon injury, the liver is capable of substantial regeneration from the original tissue until an appropriate functional size. The underlying mechanisms controlling the liver regeneration processes are not well elucidated. Previous studies have proposed that the transcription factor FoxO3 is involved in various liver diseases, but its exact role in the regulation of liver regeneration remains largely unclear. To directly test the detailed role of FoxO3 in liver regeneration, both a constitutive Albumin-Cre driver line and adeno-associated virus serotype 8 (AAV8)-Tbg-Cre (AAV-Cre)-injected adult FoxO3^fl/fl^ mice were subjected to 70% partial hepatectomy (PH). Our data demonstrate that FoxO3 deletion accelerates liver regeneration primarily by limiting polyploidization and promoting the proliferation of hepatocytes during liver regeneration. RNA-seq analysis indicates that FoxO3 deficiency greatly alters the expression of gene sets associated with cell proliferation and apoptosis during liver regeneration. Chromatin immunoprecipitation-PCR (ChIP-PCR) and luciferase reporter assays reveal that FoxO3 promotes the expression of *Nox4* but suppresses the expression of *Nr4a1* in hepatocytes. AAV8 virus-mediated overexpression of *Nox4* and knockdown of *Nr4a1* significantly suppressed hepatocyte proliferation and liver regeneration in FoxO3-deficient mice. We demonstrate that FoxO3 negatively controls hepatocyte proliferation through *Nox4* upregulation and *Nr4a1* downregulation, thereby ensuring appropriate functional regeneration of the liver. Our findings provide novel mechanistic insight into the therapeutic mechanisms of FoxO3 in liver damage and repair.

## Introduction

The liver is an organ that is capable of substantial regeneration upon injury in adult mammals^1–3^. Despite the removal of 2/3 of the liver, it can fully regrow from the remaining tissue mainly through the proliferation of hepatocytes. After 70% partial hepatectomy (PH), hepatocytes are the first cells to re-enter the cell cycle and undergo cell division until the original liver tissue is restored^1,4^. Recent studies have demonstrated that hepatocytes from the midzone of the liver lobule possess the highest capacity for proliferation during homeostasis and/or regeneration, compared with the periportal zone and central zone^5,6^. In addition, stem and progenitor cells have been proven to contribute to hepatocyte regeneration and liver tissue restoration upon injury^7–11^. These previous studies further revealed the high complexity of the mechanisms and biological pathways that govern the ability of liver regeneration after injury. Thus, it is of substantial interest to understand the detailed molecular mechanisms of liver regeneration and develop therapeutic approaches that can accelerate the regenerative process in patients with severe liver injury.

Forkhead box O (FoxO) proteins, which comprise a subfamily of forkhead transcriptional factors, play critical roles in multiple biological processes, including the cell cycle, cell proliferation, cell fate decision, and protein homeostasis^12,13^. In mammals, four members of the FoxO protein family, including FoxO1, FoxO3, FoxO4, and FoxO6, have been identified. Although these four members have overlapping functions, loss-of-function mutations in individual proteins result in specific phenotypes due to the regulation of distinct gene expression programs, indicating a different role for each FoxO isoform^14^. Of these four isoforms, FoxO3 has been shown to play a pivotal role in liver diseases including hepatocellular carcinoma (HCC)^15^, chronic hepatitis C^16^, and alcoholic liver pathogenesis^17^.

In adult and quiescent livers, the tumor suppressors p53 and p73 directly bind the promoter of *FoxO3* and activate its expression. During liver regeneration, however, the binding and activation of the chromatin structure of *FoxO3* are disrupted, which results in loss of *FoxO3* expression^18^. These findings indicate that FoxO3 might be involved in liver regeneration. However, its exact role in the regulation of liver regeneration remains largely unclear. To directly test the potential role of FoxO3 in liver regeneration, both a constitutive Albumin-Cre driver line (AKO) and adeno-associated virus serotype 8 (AAV8)-Tbg-Cre (AAV-Cre)-injected FoxO3^fl/fl^ mice with conditional loss of FoxO3 in the adult liver alone were used. Our results demonstrate that reduction in FoxO3 expression in the early stage of PH is responsible for hepatocyte proliferation and liver regeneration. Mice with liver-specific deletions of *FoxO3* exhibit accelerated liver regeneration by promoting hepatocyte proliferation, at least in part, by downregulating nicotinamide adenine dinucleotide phosphate (NADPH) oxidase 4 (*Nox4*) and upregulating nuclear orphan receptor *Nr4a1*.

## Results

### PH induces a significant decrease in FoxO3 expression in the early stage of liver regeneration

**Fig. 1:**
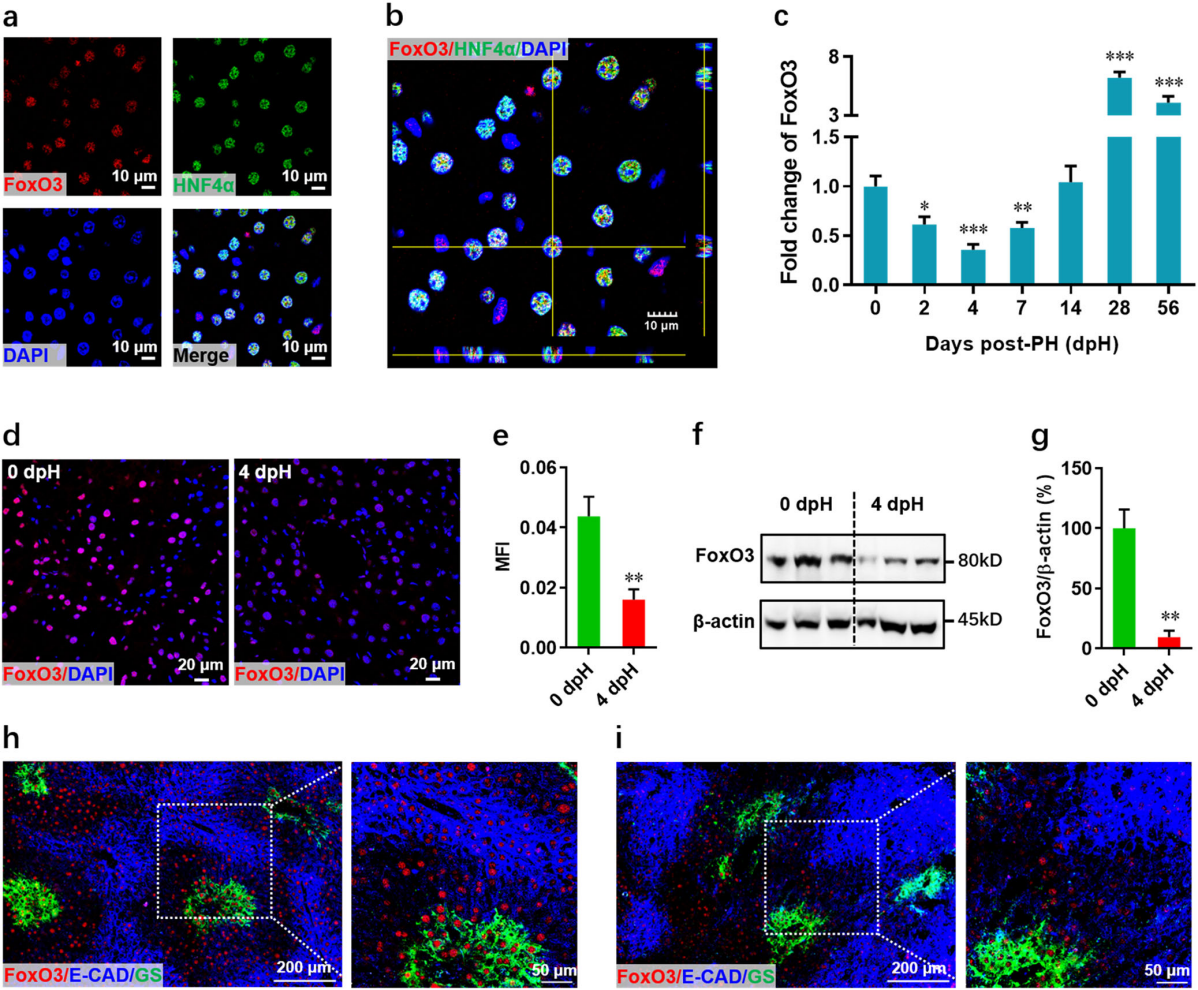
FoxO3 expression patterns during liver regeneration in wild-type mice. **a** FoxO3 expression and localization in quiescent livers of adult mice (0 dpH) was identified by staining for FoxO3 (red), HNF4α (green), and nuclei (blue). **b** Representative Z-stack confocal image of FoxO3-positive hepatocytes in liver. **c** Quantification of hepatic *FoxO3* expression at the indicated time points after PH. Data are presented as the mean ± SEM (*n* = 5 ~ 8 mice per group), *p < 0.05, **p < 0.01, ***p < 0.001 versus quiescent livers at 0 dpH (one-way ANOVA test). (d and e) The expression and localization of FoxO3 in liver were detected by immunofluorescence staining in pre-PH and 4 dpH mice. (d) Representative images for each condition are shown. **e** Quantification of FoxO3 fluorescence intensity was performed as described in the Methods section. Data are presented as the mean ± SEM (*n* = 4 mice per group), ***p < 0.001 (Student’s *t*-test). **f**, **g** Representative images of Western blotting (**f**) and relative quantification (**g**) of FoxO3 protein levels in primary hepatocytes isolated from wild-type mice at pre-PH and 4 dpH. **h**, **i** Confocal staining of FoxO3, E-CAD (E-cadherin), and GS (glutamine synthetase) revealed a zone-independent expression manner of FoxO3 in livers at 0 dpH (**h**) and 4 dpH (**i**). Data are presented as the mean ± SEM (*n* = 3 mice per group). **p < 0.01 (Student’s *t*-test).

FoxO3 was predominantly detected in hepatocyte nuclei in quiescent livers from adult mice (Fig. 1a, b), suggesting the functional importance of FoxO3 in hepatocytes. At the early stage of liver regeneration, *FoxO3* mRNA expression gradually and significantly decreased and reached a minimum level at 4 days post-PH (dpH) followed by gradual increases from 7 to 14 dpH and a remarkable elevation from 28–56 dpH (Fig. 1c). Consistent with these results, immunofluorescent staining revealed that there is a marked decrease in the FoxO3 protein level in the nuclei of liver cells at 4 dpH compared with quiescent livers at 0 dpH (pre-PH) (Fig. 1d, e). Moreover, the decreased FoxO3 protein level was further confirmed in primary hepatocytes isolated from livers with PH by western blotting (Fig. 1f, g). Therefore, the minimum level of FoxO3 expression detected at 4 dpH might imply functional importance for FoxO3 in the regulation of liver regeneration. Given that hepatocytes from different liver zones have different proliferative capacity^5,6^, we further examined the expression patterns of FoxO3 in three liver zones using the staining of E-cadherin (zone 1 marker) and glutamine synthetase (GS) (zone 3 marker). Our data showed that FoxO3 uniformly expressed in hepatocytes between three different liver zones in quiescent and regenerating livers, which implying that FoxO3 expression in hepatocytes is liver zone-independent (Fig. 1h, i).

### Constitutive loss of FoxO3 in the liver developmentally limits polyploidization and promotes hepatocyte proliferation

**Fig. 2:**
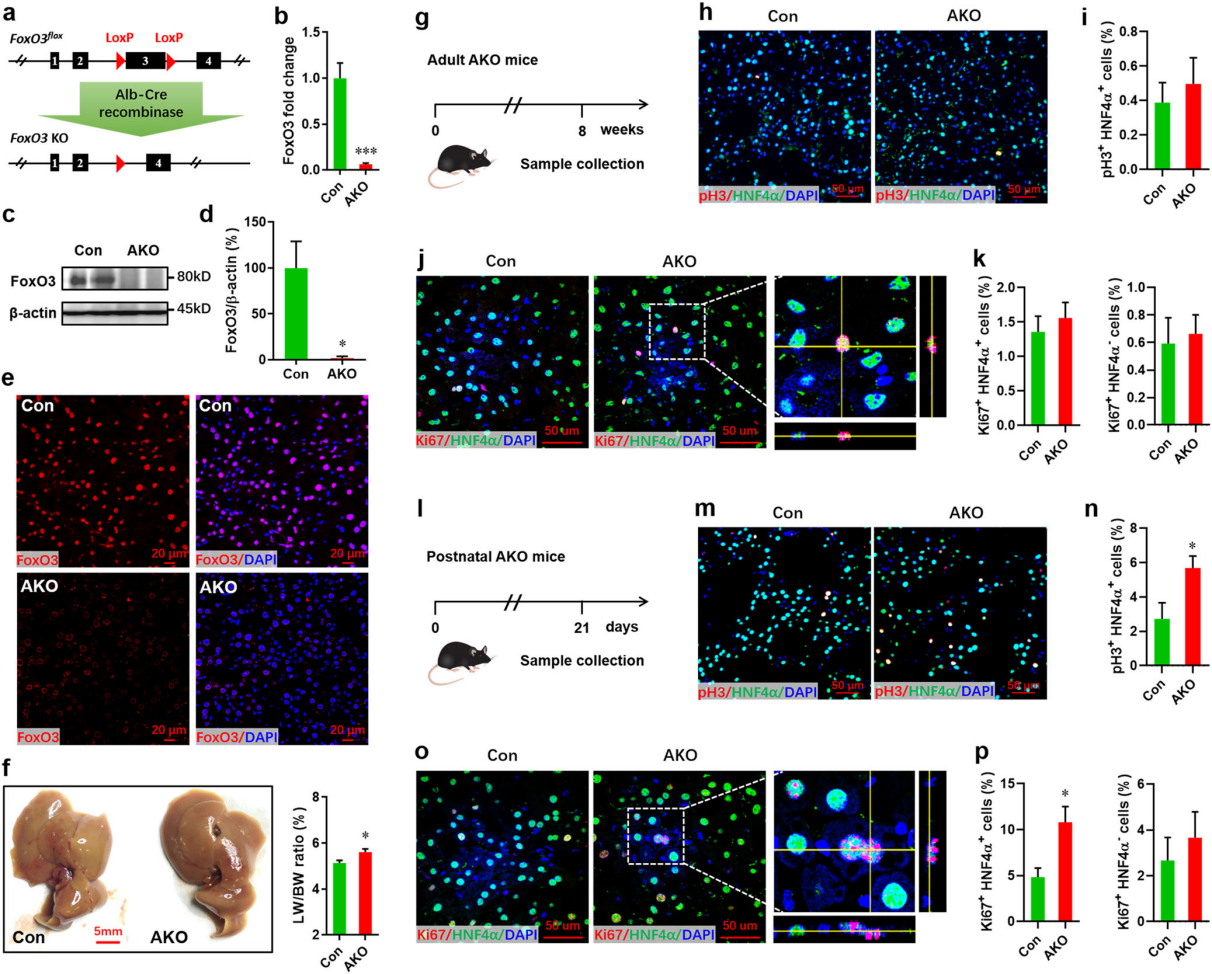
Effects of the constitutive loss of FoxO3 on proliferation of hepatocytes. **a** *FoxO3*^*fl/fl*^ mice were crossed with mice Alb-Cre mice to generate AKO mice with constitutive deletion of FoxO3 in hepatocytes. **b** qPCR validation of FoxO3 expression in livers in adult mice at 8 weeks of age (*n* = 5 mice). **c**, **d** Representative western blot images (**c**) and quantification (**d**) of FoxO3 expression in livers. **e** FoxO3 knockout in protein levels confirmed by immunofluorescence staining. **f** Representative liver images (left) and quantification of liver/body weight ratio (right) in control and AKO mice at 8 weeks of age (*n* = 7 ~ 8 mice). **g** Schematic of sample collection in adult mice at 8 weeks of age. **h** and **i** Representative images (**h**) and quantification (**i**, pH3^+^ HNF4α^+^ cells versus HNF4α^+^ cells) of pH3-positive hepatocytes in adult mice (*n* = 5 mice). **j**, **k** Representative images (**j**) and quantification of Ki67^+^ HNF4α^+^ (**k**, left) as well as Ki67^+^ HNF4α^−^ (**k**, right) cells in adult mice (*n* = 5 mice). **l** Schematic of sample collection in postnatal mice at p21. **m**, **n** Representative images (**m**) and quantification (**n**) of pH3^+^ HNF4α^+^ cells in postnatal mice (*n* = 5 mice). **o**, **p** Representative images (**o**) and quantification of Ki67^+^ HNF4α^+^ (**p**, left) as well as Ki67^+^ HNF4α^+^ (**p**, right) cells in postnatal mice (*n* = 5 mice). All data are presented as the mean ± SEM. *p < 0.05, ***p < 0.001 versus controls (Student’s *t*-test).

To confirm the influence of FoxO3 on hepatocyte proliferation, a hepatocyte-specific knockout mouse (AKO) model was generated by intercrossing *FoxO3*-floxed (*FoxO3*^*fl/fl*^) mice^19^ with Albumin-Cre transgenic (Alb-Cre) mice (Fig. 2a). In adult AKO mice, FoxO3 knockout in liver tissue and hepatocytes (Fig. 2b–e) did not influence the liver morphology or structure (Fig. 2f, left panel, and Supplementary Fig. 1). The liver/body weight ratio was slightly increased in AKO mice compared with controls (Fig. 2f, right). β-catenin staining revealed that the hepatocyte size was smaller in AKO mice (Supplementary Fig. 2a, b). Consistent with these results, an increased number of hepatocytes was detected in AKO mice (Supplementary Fig. 2c). The decreased size of primary hepatocytes isolated from AKO mice was also confirmed by flow cytometry, which demonstrated decreased values for FSC (Supplementary Fig. 2d, e). These findings suggest that there are developmental effects associated with FoxO3 deficiency, perhaps on ploidy. As expected, more mononuclear and diploid hepatocytes were detected in adult AKO mice compared with controls (Supplementary Fig. 2f-i). Surprisingly, both pH3/HNF4α and Ki67/HNF4α double staining demonstrated no significant difference in hepatocyte proliferation between adult AKO and control mice (Fig. 2g–k). Given that there was a wave of hepatocyte proliferation in development from neonatal to adult mice^3,20^, we further examined hepatocyte proliferation in younger mice at postnatal day 21 (p21). Indeed, an increased level of hepatocyte proliferation was detected in AKO mice (Fig. 2l–p). In addition, greatly increased proliferation of total liver cells was detected in AKO postnatal mice (Supplementary Fig. 3). However, there was no significant difference in the proliferation of non-hepatocytes (HNF4α^−^ Ki67^+^) between AKO and control neonatal mice (Fig. 2p, right). Therefore, these findings imply that constitutive loss of FoxO3 developmentally promotes the proliferation of hepatocytes and reduces the polyploidization of hepatocytes.

### Effects of FoxO3 on hepatocyte proliferation in vitro

To further determine the effects of FoxO3 on hepatocyte proliferation in vitro, a stable *FoxO3*-knockdown NCTC1469 cell line (a murine liver cell line) was established using lentiviral-mediated shRNA (shFoxO3). Knockdown efficiency was confirmed at the mRNA and protein levels (Supplementary Fig. 4a, b). Cell proliferation was evaluated by a nuclear incorporation assay using 5-Ethynyl-2'-deoxyuridine (EdU). The percentage of EdU^+^ cells and the total cell number were significantly elevated by FoxO3 knockdown (Supplementary Fig. 4c–e). Subsequently, a FoxO3-overexpressing cell line was established and confirmed by real-time quantitative PCR (qPCR) (Supplementary Fig. 4f). In contrast with the knockdown cells, FoxO3 overexpression remarkably suppressed the proliferation of NCTC1469 cells as demonstrated by nuclear incorporation of EdU and total cell number assessment (Supplementary Fig. 4g–i). These findings reveal that hepatocyte proliferation is negatively regulated by FoxO3.

### Constitutive loss of FoxO3 promotes hepatocyte proliferation and restricts polyploidization during liver regeneration

**Fig. 3:**
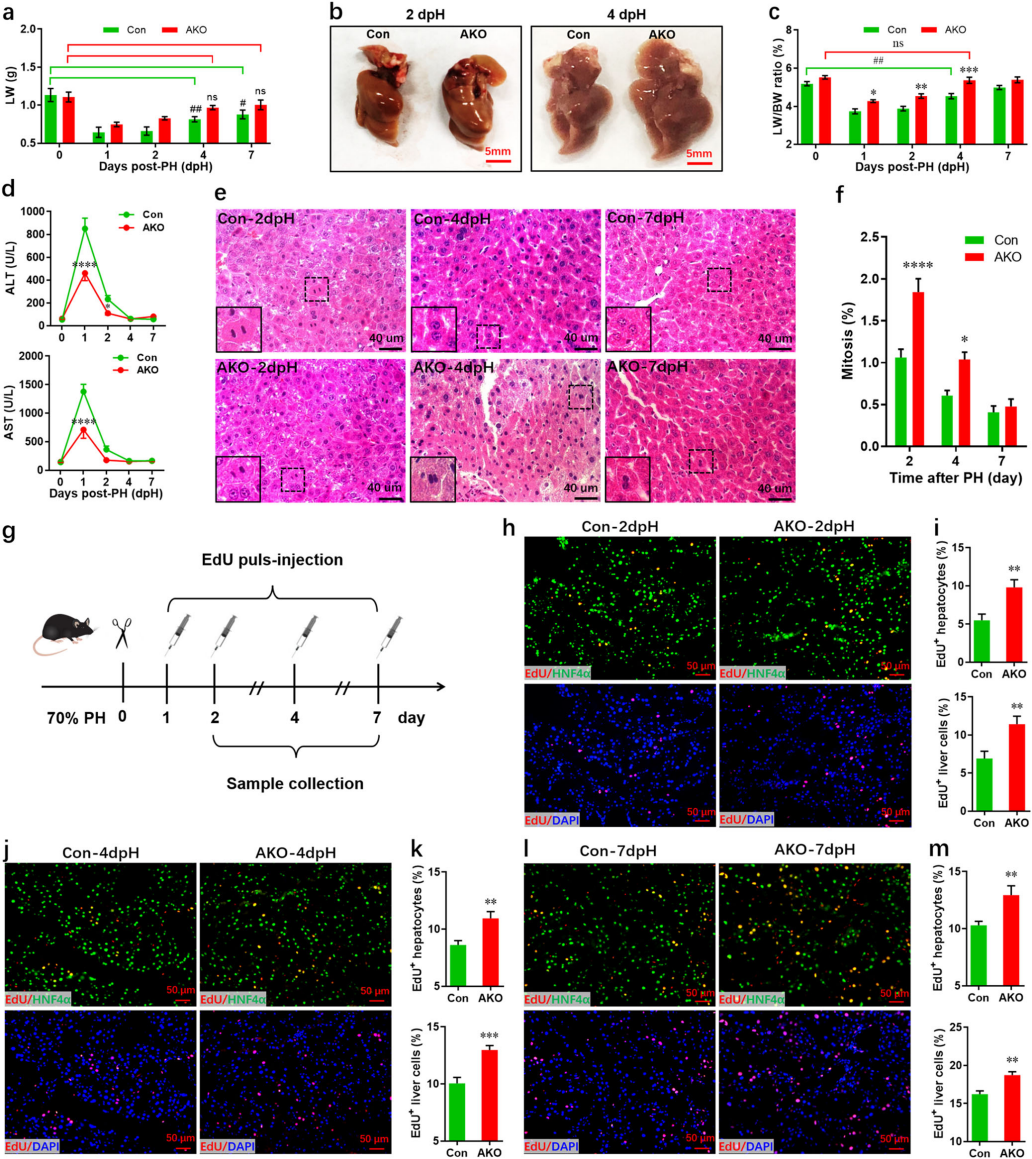
Enhanced liver regeneration and hepatocyte proliferation in AKO mice after PH. **a** Recovery of liver weight in control and AKO mice after PH (*n* = 5 ~ 8 mice per group). **b** Representative images of livers from control and AKO mice at 2 and 4 dpH. **c** The actual ratio of liver weight to body weight at the indicated time points after PH (*n* = 5 ~ 8 mice per group). **d** Serum AST (upper) and ALT (lower) levels in control and *FoxO3*-knockout mice pre- and post-PH (*n* = 6 mice per group). **e** Representative liver sections stained with hematoxylin and eosin showing mitotic changes in hepatocytes in control and AKO mice after PH. **f** Quantification of mitotic hepatocytes in control and AKO mice (*n* = 5 mice per group). **g** Schematic of an EdU pulse-chase experiment designed to label proliferating hepatocytes during liver regeneration. **h**, **i** Representative images (**h**) and quantification (**i**) of EdU^+^ hepatocytes (upper) and liver cells (lower) in control and AKO mice at 2 dpH (*n* = 8 mice per group). **j**, **k** Representative images (**j**) and quantification (**k**) of EdU^+^ hepatocytes (upper) and liver cells (lower) in control and AKO mice at 4 dpH (*n* = 8 mice per group). **l**, **m** Representative images (**l**) and quantification (**m**) of EdU^+^ hepatocytes (upper) and liver cells (lower) in control and AKO mice at 7 dpH (*n* = 8 mice per group). All data are presented as the mean ± SEM. *p < 0.05, **p < 0.01, ***p < 0.001 versus control mice; ^#^*p* < 0.05; ns, no significant difference (two-way ANOVA test for **a**, **c**, **d**, **f**, and Student’s *t*-test for **i**, **k**, and **m**).

To further investigate the in vivo role of FoxO3 in liver regeneration, we performed PH on adult AKO and control mice and analyzed liver/body weight ratio. Elevated liver weight was detected in AKO mice during regeneration (Fig. 3a), suggesting that FoxO3 deficiency promotes liver regeneration at the early stage of PH. Indeed, liver images revealed an increase in liver recovery in AKO mice at 2 and 4 dpH (Fig. 3b). Consistent with these findings, the actual liver/body weight ratio was higher for AKO mice compared with controls during the first 4 days post-PH. Importantly, the liver/body weight ratio at 4 dpH reached the level prior to PH in AKO mice, whereas the level for control mice at 4 dpH was significantly lower than that at 0 PH (Fig. 3c). In line with these results, FoxO3 deficiency reduced liver damage as demonstrated by the decreased activity of serum ALT and AST at 1 and 2 dpH (Fig. 3d). Therefore, it is likely that FoxO3 deficiency accelerates liver regeneration after PH.

Next, we examined the in vivo proliferation of hepatocytes in AKO and control mice during liver regeneration. Notably, the mitotic activity was significantly enhanced in AKO mice at 2 and 4 dpH compared with controls (Fig. 3e and f). Consistent with these results, EdU pulse-chase assay (Fig. 3g) demonstrated that the percentage of EdU^+^ hepatocytes greatly increase in AKO mice at 2 dpH compared with controls (Fig. 3h and i, upper panel). Consistent with these results, a remarkable increase in the proliferation level of total liver cells was detected in AKO mice (Fig. 3h and i, lower panel). Moreover, increased proliferation of hepatocytes and total liver cells in AKO mice was detected at 4 and 7 dpH after EdU pulse-chase injections (Fig. 3j–m).

To confirm these results, hepatocyte proliferation was further assessed using other proliferative markers including pH3, proliferating cell nuclear antigen (PCNA), and Ki67. In agreement with the EdU incorporation assay, pH3/HNF4α double staining revealed that FoxO3 deficiency enhances hepatocyte proliferation at 2 and 4 dpH (Supplementary Fig. 5a, b). The elevated hepatocyte proliferation in AKO mice was further confirmed by proliferating cell nuclear antigen (PCNA)/HNF4α double staining (Supplementary Fig. 5c, d). In addition, the increased proliferation of hepatocytes in AKO mice was also evidenced by Ki67/HNF4α double staining, despite no difference in the proliferation of nonparenchymal cell (HNF4α^-^ Ki67^+^ cells) (Supplementary Fig. 5e–g). To explore whether and how FoxO3-deficient hepatocytes respond to primary hepatocyte mitogens such as EGF and HGF, primary hepatocytes isolated from adult AKO and control mice were subjected to hepatocyte growth assays^21^. There was no significant difference in the percentage of EdU^+^ hepatocytes between AKO and control mice without mitogen stimulation. However, FoxO3 deficiency synergistically promoted hepatocyte proliferation induced by mitogens (Supplementary Fig. 6a, b). Thus, these findings suggest that FoxO3-deficient hepatocytes are more sensitive to primary hepatocyte mitogens. To further explore the potential underlying mechanism, primary hepatocytes isolated from control and AKO adult mice were subjected to Western blotting assays to evaluate the expression of the EGF receptor (EGFR) and HGF receptor (MET). Our data demonstrated that FoxO3 deficiency had no significant effects on the expression of EGFR and MET (Supplementary Fig. 6c, d). These results suggest that the higher sensitivity of FoxO3-deficient hepatocytes to mitogens may not result from the higher expression of mitogen receptors.

**Fig. 4:**
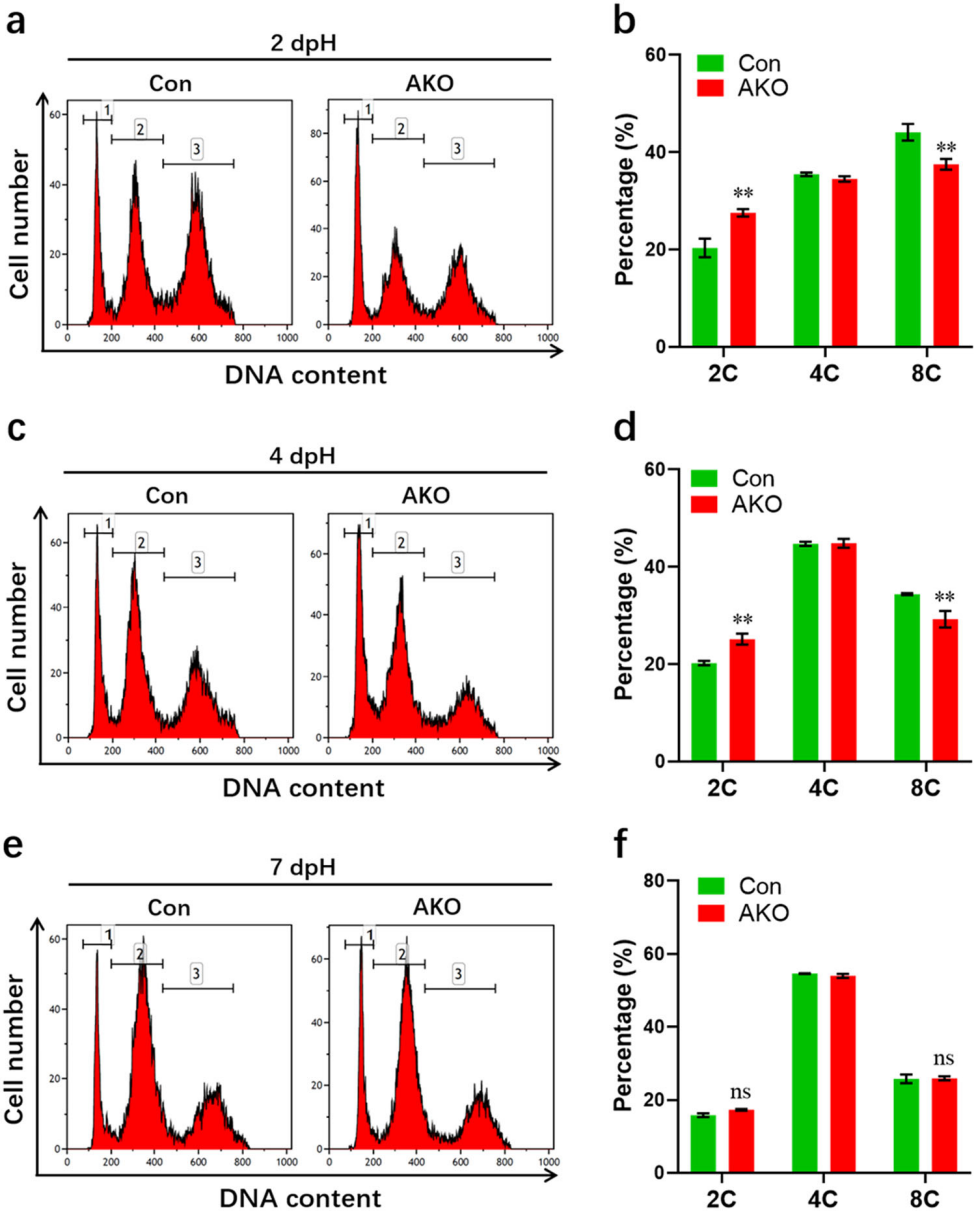
Effects of constitutive loss of FoxO3 on hepatocyte ploidy during liver regeneration in adult mice. Primary hepatocytes isolated from livers at 2 (**a**, **b**), 4 (**c**, **d**), and 7 (**e**, **f**) dpH were subjected to ploidy contribution analysis using flow cytometry. Representative images (**a**, **c**, and **e**) and quantification (**b**, **d**, and **f**) of the ploidy contribution of hepatocytes isolated from Con and AKO mice are shown (*n* = 3 mice per group). Peaks 1, 2 and 3 depicted, respectively, 2 C, 4 C, and 8 C cells. All data are presented as the mean ± SEM. **p < 0.01 versus control mice (two-way ANOVA test).

Consistent with these results, increased diploid and decreased polyploid hepatocytes were detected in AKO mice in the early stage of PH when compared with controls (Fig. 4). In addition, decreased hepatocyte size and an increased number of liver cells were observed in regenerating liver tissues in AKO mice as demonstrated by β-catenin staining assays (Supplementary Fig. 7). The decreased size of primary hepatocytes isolated from AKO mice was further confirmed by flow cytometry as validated by decreased FSC values (Supplementary Fig. 8). These findings indicate that constitutive loss of FoxO3 promotes proliferation and restricts the polyploidization of hepatocytes, thereby encouraging acute liver regeneration in adult mice. Moreover, FoxO3 deficiency suppresses the apoptosis of liver cells in the early stage of PH (Supplementary Fig. 9). Given that p53 is a binding protein for FoxO3, the expression of p53 was investigated during liver regeneration. Our data demonstrated that loss of FoxO3 had no significant influence on the p53 protein level in AKO and control livers at 2 dpH (Supplementary Fig. 10), suggesting that p53 might not be regulated by FoxO3 during liver regeneration.

### Conditional loss of FoxO3 in adult livers alone promotes hepatocyte proliferation during regeneration

**Fig. 5:**
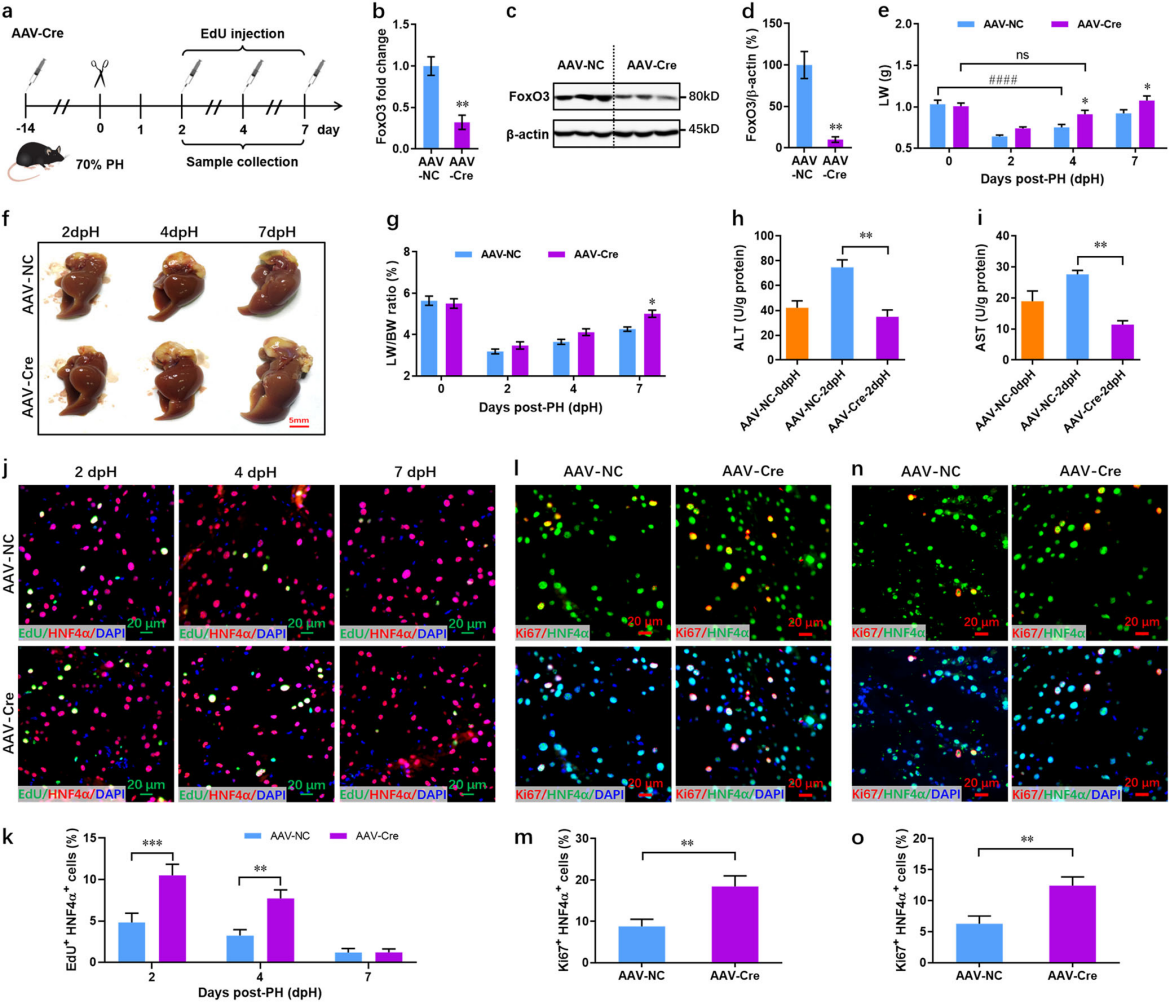
Conditional loss of FoxO3 only in adult mice promotes liver regeneration via hepatocyte proliferation. **a** Schematic of AAV-Cre injection in adult FoxO3^fl/fl^ mice followed by PH performance and EdU injection at individual time points. **b** qPCR validation of FoxO3 expression in livers from AAV-Cre and control mice (*n* = 3 mice). **c**, **d** Representative western blot images (**c**) and quantification (**d**) of FoxO3 expression in primary hepatocytes. **e** Recovery of liver weight in control and AAV-Cre mice after PH (*n* = 10 mice per group). **f** Representative images of livers from control and AAV-Cre mice at 2-7 dpH. **g** The actual ratio of liver weight (LW) to body weight (BW) at the indicated time points after PH (*n* = 10 mice per group). **h**, **i** Liver AST (**h**) and ALT (**i**) levels in control and AAV-Cre mice pre- and post-PH (*n* = 3 mice per group). **j**, **k** Representative images (**j**) and quantification (**k**) of EdU^+^ HNF4α^+^ cells in control and AAV-Cre mice at indicated time points after PH (*n* = 8 mice per group). **l**, **m** Representative images (**l**) and quantification (**m**) of Ki67^+^ HNF4α^+^ cells in control and AAV-Cre mice at 2 dpH (*n* = 8 mice per group). **n**, **o** Representative images (**n**) and quantification (**o**) of Ki67^+^ HNF4α^+^ cells in control and AAV-Cre mice at 4 dpH (*n* = 8 mice per group). All data are presented as the mean ± SEM. *p < 0.05, **p < 0.01, ***p < 0.001 versus control mice; ^###^*p* < 0.001; ns, no significant difference (Student’s *t*-test for **b**, **d**, **m**, and **o**; two-way ANOVA tes*t* for **e**, **g**, and **k**; one-way ANOVA test for **h** and **i**).

To further separate and explore the primary cause of faster liver regeneration in FoxO3 null mice, adult FoxO3^fl/fl^ mice were injected with AAV8-Tbg-Cre (AAV-Cre) 2 weeks prior to PH. The ideal concentration of AAV-Cre were confirmed using a reporter mouse strain (Supplementary Fig. 11). These AAV-Cre-injected FoxO3^fl/fl^ mice were then subjected to PH and EdU labeling at individual time points (Fig. 5a). Loss of FoxO3 in the adult liver induced by AAV-Cre was confirmed by qPCR assay at 0 dpH (Fig. 5b). Furthermore, loss of FoxO3 was further detected in primary hepatocytes isolated from adult mice with AAV-Cre injection at 0 dpH (Fig. 5c, d). However, the loss of FoxO3 did not influence the ploidy and size of hepatocytes at 0 dpH (Supplementary Fig. 12a–d). Both pH3/HNF4α and Ki67/HNF4α double staining revealed that there were no significant differences in hepatocyte proliferation at 0 dpH between the AAV-Cre and control viruses (Supplementary Fig. 12e–h). In line with the constitutive deletion in AKO mice, AAV-Cre-induced deletion of FoxO3 promoted liver growth within 1 week after PH. The liver weight in mutant mice recovered as early as 4 dpH, whereas the liver weight in AAV-NC mice did not reach the same level of quiescent livers until 7 dpH (Fig. 5e, f). Furthermore, the liver/body weight ratio was elevated for AAV-Cre compared with AAV-NC mice (Fig. 5g). Consistent with these results, the PH-induced increases in the ALT and AST levels in liver tissues were suppressed by AAV-Cre viruses at 2 dpH (Fig. 5h, i).

Moreover, conditional loss of FoxO3 in the adult liver alone significantly increased the percentage of HNF4α^+^ EdU^+^ cells at 2 and 4 dpH (Fig. 5j, k). Ki67/HNF4α double staining further revealed that the proliferation of hepatocytes was remarkably elevated in AAV-Cre mice at 2 and 4 dpH (Fig. 5l–o). Using the CCl4-induced liver injury model, we further confirmed that conditional loss of FoxO3 in the adult liver alone indeed increased the proliferation of hepatocytes (Supplementary Fig. 13). However, conditional loss of FoxO3 in the adult liver alone did not influence the ploidy and size of hepatocytes at 2 and 4 dpH (Supplementary Fig. 14). We also used a 30% PH injury model to assess hepatocyte size during liver regeneration using the constitutive and conditional knockout mice. Our results demonstrated that constitutive loss of FoxO3 reduced hepatocyte size upon 30% PH injury (Supplementary Fig. 15a–c). However, conditional loss of FoxO3 specifically in adult mice did not influence the hepatocyte size during liver regeneration in the 30% PH injury model (Supplementary Fig. 15d–f). To determine whether conditional loss of FoxO3 influences hepatocyte homeostasis, adult FoxO3^fl/fl^ mice were injected with AAV-Cre and control viruses for 4 weeks. Our data demonstrated that there were no significant differences in hepatocyte size between the AAV-Cre and AAV-NC groups during the 4 weeks after virus injection (Supplementary Fig. 16). Therefore, these findings indicate that conditional loss of FoxO3 promotes acute liver regeneration, primarily depending on an increase in proliferation of hepatocytes.

### Variations in FoxO3-regulated genes in livers upon PH

**Fig. 6:**
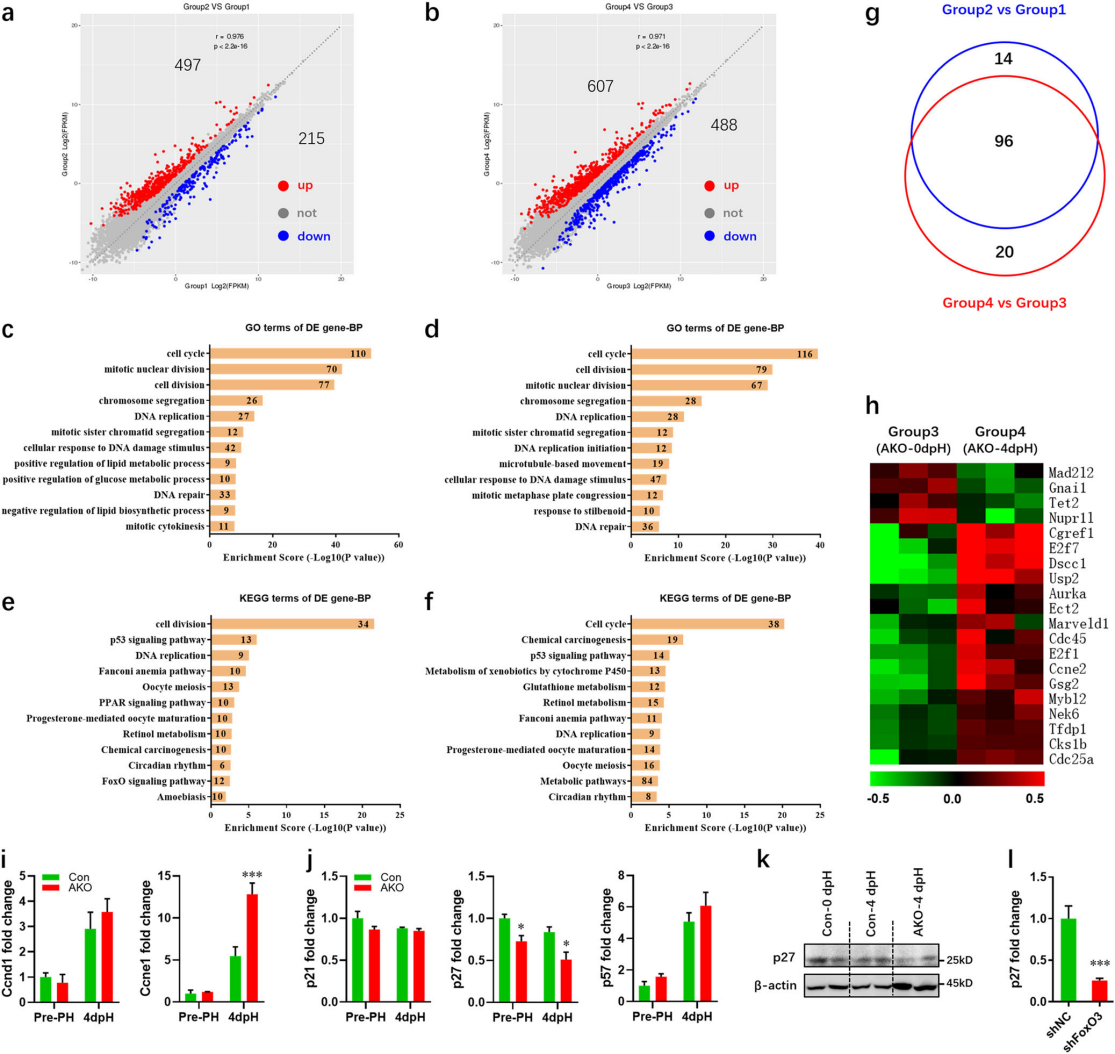
Cell cycle-related genes are critical to liver regeneration in AKO mice. **a**, **b** Overall changes of genes in livers from control (**a**) and *FoxO3*-knockout (**b**) mice at 4 dpH compared with 0 dpH. Group1, livers from control mice before PH; Group2, livers from control mice 4 days after PH; Group3, livers from knockout mice before PH; Group4, livers from knockout mice 4 days after PH. **c**, **d** GO analysis of differentially expressed genes in livers from control (**c**) and FoxO3-knockout (**d**) mice at 4 dpH compared with 0 dpH. **e**, **f** KEGG pathway analysis of differentially expressed genes in livers from control (**e**) and FoxO3-knockout (**f**) mice at 4 dpH compared with 0 dpH. **g** Venn diagrams showing overlap of the altered cell cycle-related genes between control and *FoxO3*-knockout livers. **h** Heat map of the cell cycle-related genes isolated specifically from FoxO3-knockout livers. **i** Quantification of cyclin D1 (left) and E1 (right) in control and *FoxO3*-knockout livers pre- and post-PH (*n* = 3 mice per group). **j** Quantification of cyclin dependent kinase inhibitors (p21, p27, and p57) in control and *FoxO3*-knockout livers pre- and post-PH (*n* = 3 mice per group). **k** Activation of p27 in control and AKO livers at 4 dpH was analyzed using western blotting. **l** qPCR validation of *p27* expression in NCTC1469 cells with and without *FoxO3* silencing (*n* = 6 per group). All data are presented as the mean ± SEM, *p < 0.05, ***p < 0.001 versus controls (two-way ANOVA test for **i** and **j**; Student’s *t*-test for **l**).

To explore the gene expression patterns during regeneration, liver tissues from AKO and control mice at 4 dpH were subjected to RNA-seq analysis. We identified 497 upregulated and 215 downregulated genes in control mice at 4 dpH compared with pre-PH (Fig. 6a). For mutant mice, 607 upregulated and 488 downregulated genes were identified in injured livers (Fig. 6b). Gene ontology (GO) and KEGG pathway enrichment analysis revealed that the PH-induced differentially expressed genes in wild-type and mutant mice were significantly enriched for cell cycle-related gene sets where 20 genes were specifically identified in FoxO3-deficient mice (Fig. 6c–h). We found that cyclin E1 expression is increased in FoxO3-knockout livers at 4 dpH compared with control livers (Fig. 6i). Furthermore, decreased p27 expression was detected in FoxO3-deficient livers during regeneration (Fig. 6j, k). These data were further confirmed in hepatocytes with FoxO3 deficiency (Fig. 6l). In addition, 109 differentially expressed genes were identified in FoxO3-deficient mice prior to PH compared with control mice (Supplementary Fig. 17a). These genes were significantly enriched for GO terms associated with metabolism (Supplementary Fig. 17b). Upon PH, 105 differentially expressed genes were found when comparing mutant to control mice, and these were enriched for GO terms associated with apoptosis and proliferation (Supplementary Fig. 17c, d).

### Upregulated Nr4a1 contributes to hepatocyte proliferation in FoxO3-deficient mice

**Fig. 7:**
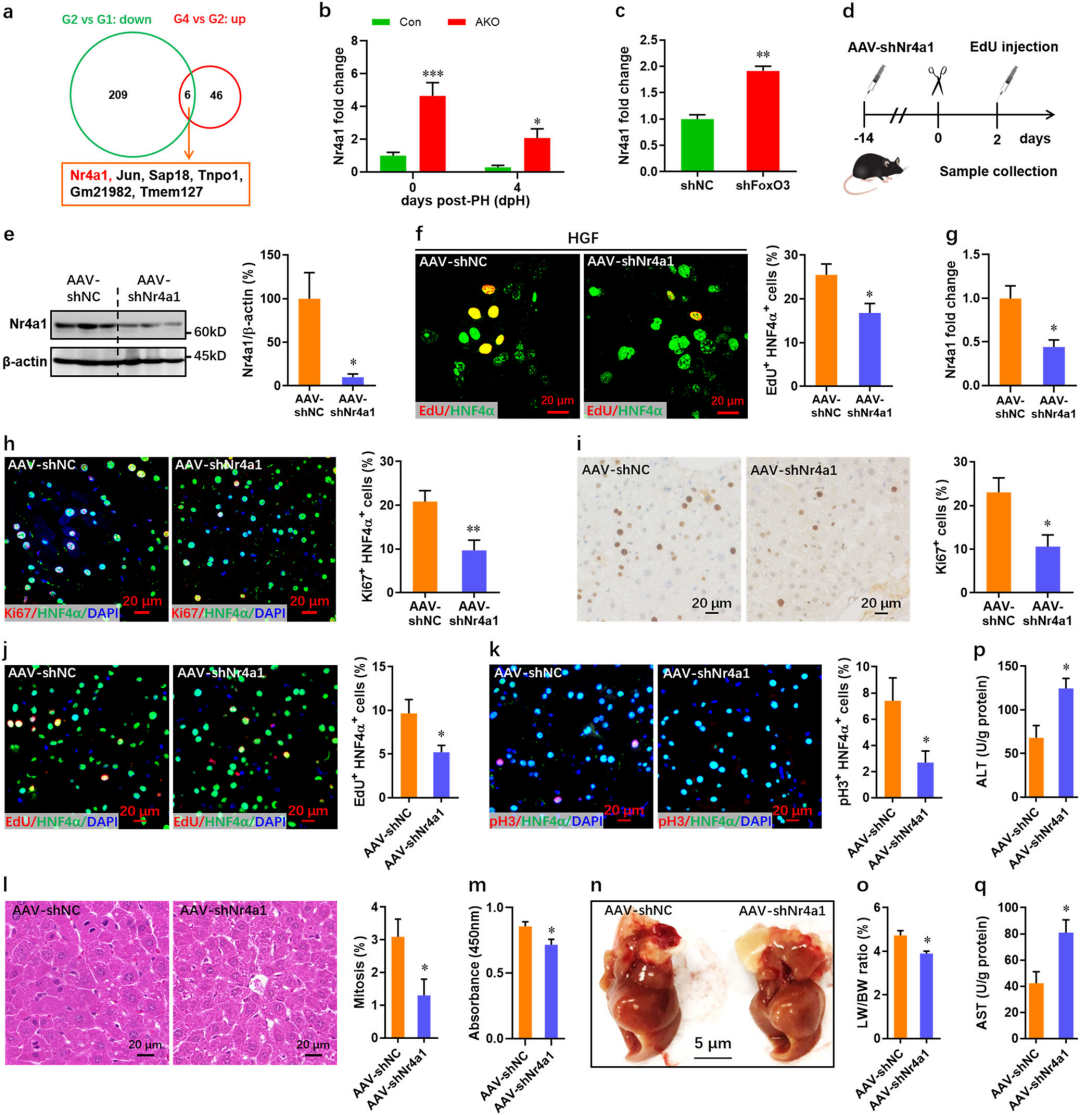
Knockdown of *Nr4a1* inhibits the proliferation of FoxO3-deficient hepatocytes. **a** RNA-seq identified six overlapping genes between the downregulated gene sets in control mice post-PH (G2) versus pre-PH (G1) and upregulated gene sets in AKO mice (G4) versus control mice post-PH (G2). G1, control mice pre-PH; G2, control mice post-PH; G4, mutant mice post-PH. **b** qPCR validation of *Nr4a1* expression in AKO mice before and after PH (*n* = 6 mice per group). **c** Upregulation of *Nr4a1* expression in *FoxO3*-deficient NCTC1469 cells (*n* = 3 per group). **d** Schematic of AAV-shNr4a1 injection in adult AKO mice for 2 weeks, followed by primary hepatocytes isolation at 0 dpH or PH performance and EdU injection at 2 dpH. **e**, **f** Isolated primary hepatocytes at 0 dpH were treated with HGF (40 ng/ml) for 4 days in MHGM, followed by Western blotting assay (e, *n* = 6 per group) and EdU/HNF4α double staining (**f**, *n* = 6 per group). **g** qPCR validation of *Nr4a1* expression in livers at 2 dpH (*n* = 4 mice). **h** Representative images (left) and quantification (right) of Ki67^+^ HNF4α^+^ cells in liver at 2 dpH (*n* = 6 mice per group). **i** Representative images (left) and quantification (right) of Ki67^+^ cells in liver at 2 dpH (*n* = 6 mice per group). **j** Representative images (left) and quantification (right) of EdU^+^ HNF4α^+^ cells in AAV-shNC and AAV-shNr4a1 mice at 2 dpH (*n* = 6 mice per group). **k** Representative images (left) and quantification (right) of pH3^+^ HNF4α^+^ cells in livers at 2 dpH (*n* = 6 mice per group). **l** Representative images (left) and quantification (right) of mitotic hepatocytes in livers at 2 dpH (*n* = 6 mice per group). **m** Cell counting assay for primary hepatocytes isolated from livers at 2 dpH (*n* = 6 mice per group). **n** Representative images of livers at 2 dpH. **o** The actual ratio of liver/body weight at 2 dpH (*n* = 6 mice per group). **p**, **q** Liver ALT (**p**) and AST (**q**) levels in AKO mice injected with AAV-shNC and AAV-shNr4a1 at 2 dpH (*n* = 3 mice per group). All data are presented as the mean ± SEM. *p < 0.05, **p < 0.01, ***p < 0.001 versus controls (two-way ANOVA test for **b**; Student’s *t*-test for **c**, **e**–**q**).

To further identify potential target genes involved in accelerated liver regeneration in FoxO3-deficient mice, association analysis of the genes differentially expressing between control and mutant mice was performed. RNA-seq analysis identified 215 downregulated genes post-PH in control mice, and 6 of these genes overlapped with genes upregulated in mutant compared with control mice at 4 dpH (Fig. 7a). Among the 6 overlapping genes, only *Nr4a1* was significantly and consistently upregulated by FoxO3 deficiency both pre- and post-PH (Fig. 7b and Supplementary Fig. 18). Negative regulation of *Nr4a1* was further demonstrated in *FoxO3*-deficient NCTC1469 cells (Fig. 7c). To elucidate whether and how FoxO3 regulates the expression of *Nr4a1*, we subsequently analyzed the promoter sequence of *Nr4a1* and identified seven potential FoxO3 binding sites (Supplementary Table 1). The binding site with the highest score was further analyzed by ChIP-qPCR, and it was used to examine the in vivo interaction between FoxO3 and the *Nr4a1* promoter in mouse liver (Supplementary Fig. 19a). Moreover, luciferase reporter assays (Supplementary Fig. 19b) revealed that *FoxO3* overexpression greatly suppressed the relative luciferase activity driven by the wild-type*Nr4a1* promoter (Supplementary Fig. 19c). However, mutation of the consensus FoxO3 binding sites attenuated the FoxO3-mediated suppression of luciferase activity (Supplementary Fig. 19c). To further elucidate whether and how Nr4a1 interfere with the proliferation of FoxO3-deficient hepatocytes, primary hepatocytes were isolated from AKO mice at 0 dpH after injection with AAV8-shNr4a1 2 weeks prior to PH (Fig. 7d) and subjected to stimulation with HGF, followed by proliferation assay using EdU/HNF4α double staining. Our data revealed that the suppression of Nr4a1 in primary FoxO3-deficient hepatocytes (Fig. 7e) significantly decreased the percentage of EdU^+^ HNF4α^+^ cells (Fig. 7f). Consistent with these results, *Nr4a1* silencing in *FoxO3*-deficient NCTC1469 cells remarkably suppressed proliferation as demonstrated by nuclear incorporation of EdU (Supplementary Fig. 20).

To further confirm the effects of Nr4a1 on hepatocyte proliferation during liver regeneration, we further knocked down the expression of *Nr4a1* in AKO mice using AAV8-shNr4a1 and analyzed the hepatocyte proliferation and liver regeneration at 2 dpH (Fig. 7d). In vivo knockdown of *Nr4a1* (Fig. 7g) significantly decreased the percentage of Ki67^+^ and EdU^+^ hepatocytes in AKO mice (Fig. 7h–j). Moreover, pH3/HNF4α double staining (Fig. 7k) and mitotic activity assays (Fig. 7l) further confirmed the decreased proliferation of hepatocytes induced by Nr4a1 knockdown in AKO mice. In addition, cell counting assays revealed decreased proliferation in primary hepatocytes isolated from AAV8-shNr4a1-injected mice at 2 dpH (Fig. 7m). In line with these results, the actual liver/body weight ratio was suppressed by Nr4a1 knockdown in AKO mice at 2 dpH (Fig. 7n, o). Moreover, Nr4a1 knockdown results in increased levels of ALT and AST in livers (Fig. 7p, q).

### Nox4 is critical for FoxO3-regulated hepatocyte proliferation and apoptosis during regeneration

**Fig. 8:**
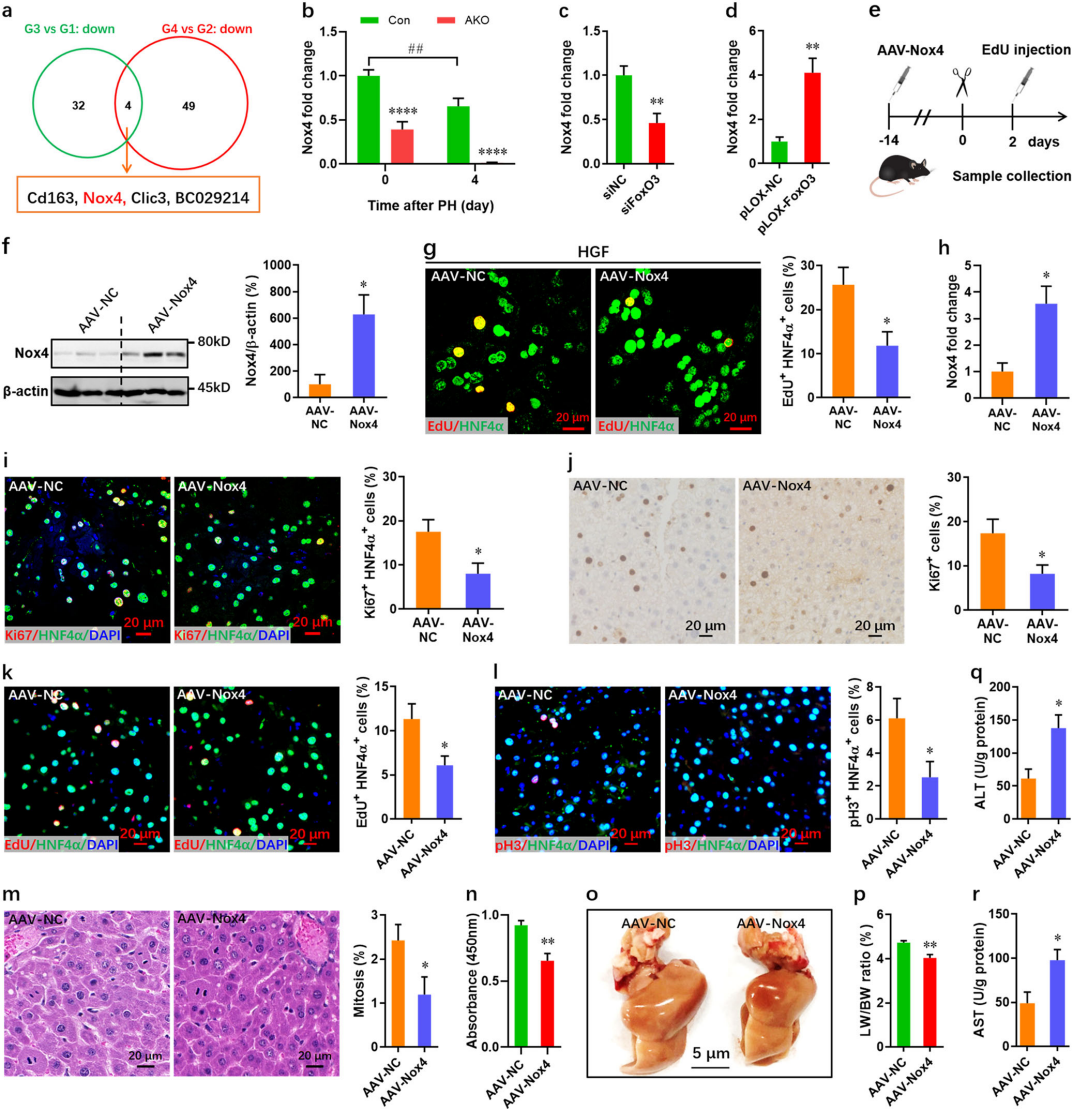
FoxO3 deficiency-induced hepatocyte proliferation exhibits a strong correlation with Nox4 downregulation. **a** RNA-seq identified 4 overlapping downregulated genes in AKO mice pre- and post-PH compared with control mice. G1, control mice pre-PH; G2, control mice post-PH; G3, mutant mice pre-PH; G4, mutant mice post-PH. **b** qPCR validation of the downregulated expression of *Nox4* in *FoxO3*-knockout mice compared with control mice before and after PH (*n* = 8 mice per group). **c** Downregulation of *Nox4* expression in *FoxO3*-deficient NCTC1469 cells (*n* = 5 per group). **d** Upregulation of *Nox4* expression in *FoxO3*-overexpressing NCTC1469 cells (*n* = 5 per group). **e** Schematic of AAV-Nox4 injection in adult AKO mice for 2 weeks, followed by primary hepatocytes isolation at 0 dpH or PH performance and EdU injection at 2 dpH. **f**, **g** Isolated primary hepatocytes at 0 dpH were treated with HGF (40 ng/ml) for 4 days in MHGM, followed by Western blotting assay (f, *n* = 3 mice) and EdU/HNF4α double staining (g, *n* = 6 mice). **h** qPCR validation of *Nox4* expression in livers from AAV-NC and AAV-Nox4 mice (*n* = 3 mice). **i** Representative images (left) and quantification (right) of Ki67^+^ HNF4α^+^ cells in AAV-NC and AAV-Nox4 mice at 2 dpH (*n* = 6 mice). **j** Representative images (left) and quantification (right) of Ki67^+^ cells in liver at 2 dpH (*n* = 6 mice). **k** Representative images (left) and quantification (right) of EdU^+^ HNF4α^+^ cells in AAV-NC and AAV-Nox4 mice at 2 dpH (*n* = 6 mice). **l** Representative images (left) and quantification (right) of pH3^+^ HNF4α^+^ cells in livers at 2 dpH (*n* = 6 mice). **m** Representative images (left) and quantification (right) of mitotic hepatocytes in livers at 2 dpH (*n* = 6 mice). **n** Cell counting assay for primary hepatocytes isolated from livers at 2 dpH (*n* = 6). **o** Representative images of livers at 2 dpH. **p** The actual ratio of liver/body weight at 2 dpH (*n* = 6 mice). **q** and **r** Liver ALT (**q**) and AST (**r**) levels in AKO mice injected with AAV-NC and AAV-Nox4 at 2 dpH (*n* = 3 mice). All data are presented as the mean ± SEM, *p < 0.05, **p < 0.01, ***p < 0.001 versus controls (two-way ANOVA test for b; Student’s *t*-test for c, d, f-r).

To further explore the molecular mechanism responsible for the accelerated liver regeneration in mutant mice, four overlapping genes were identified among the downregulated genes in *FoxO3*-deficient compared with wild-type mice, regardless of PH (Fig. 8a). Of these four genes, the expression of *Nox4* (Fig. 8b) and *Cd163* (Supplementary Fig. 21) was remarkably suppressed in the liver by FoxO3 deficiency, regardless of the PH. However, *Nox4* rather than *Cd163* was significantly downregulated in liver upon PH in wild-type mice (Fig. 8b and Supplementary Fig. 21), implying a greater importance for *Nox4* during liver injury and regeneration. *FoxO3* silencing in NCTC1469 cells resulted in the downregulation of *Nox4* expression by >50% (Fig. 8c). As expected, *Nox4* expression was increased 3-fold in *FoxO3*-overexpressing cells (Fig. 8d). These findings suggest that decreased *Nox4* expression might be responsible for the accelerated liver regeneration in *FoxO3*-knockout mice.

To elucidate whether and how FoxO3 regulates the expression of *Nox4* in mouse liver, we subsequently analyzed the *Nox4* promoter sequence and identified 15 potential FoxO3 binding sites (Supplementary Table 2). The binding site with the highest score was further analyzed by ChIP-qPCR, which revealed the in vivo interaction between FoxO3 and the *Nox4* promoter in mouse liver (Supplementary Fig. 22a). Using a luciferase reporter plasmid containing the *Nox4* promoter (Supplementary Fig. 22b), we demonstrated that FoxO3 can interact with the *Nox4* promoter and regulate *Nox4* expression in liver cells (Supplementary Fig. 22c).

To elucidate whether and how Nox4 interfere with the proliferation of FoxO3-deficient hepatocytes, primary hepatocytes isolated from AKO mice at 0 dpH after injection with AAV8-Tbg-Nox4 (AAV-Nox4) 2 weeks prior to PH (Fig. 8e) were treated with HGF stimulation. Our data revealed that overexpression of Nox4 in primary FoxO3-deficient hepatocytes (Fig. 8f) significantly decreased the percentage of EdU^+^ HNF4α^+^ cells (Fig. 8g). In addition, the proliferation of FoxO3-overexpressing NCTC1469 cells was further elevated by *Nox4* knockdown (Supplementary Fig. 23a–e). To determine whether Nox4 expression is influenced by Nr4a1, we assessed the expression of Nox4 in NCTC1469 cells transfected with siNr4a1. Our data revealed that Nr4a1 inhibition has no significant effects on the expression of Nox4 (Supplementary Fig. 23f). These data suggest that downregulated Nox4 indeed contributes to the increased proliferation of hepatocytes in AKO mice. To further confirm this idea in vivo, AAV-Nox4 was injected into AKO mice to overexpress *Nox4* for 2 weeks, and we then examined the proliferation of hepatocytes in the liver at 2 dpH (Fig. 8e). In vivo overexpression of Nox4 in livers (Fig. 8h) significantly suppressed the proliferation of hepatocytes in AKO mice at 2 dpH (Fig. 8i–k). Furthermore, overexpression of Nox4 decreased the percentage of pH3^+^ HNF4α^+^ cells (Fig. 8l) and reduced the mitotic activity of hepatocytes (Fig. 8m) in livers of AKO mice. Consistently, decreased proliferation of the primary hepatocytes isolated from AAV8-Nox4-injected mice at 2 dpH was also demonstrated by cell counting assay (Fig. 8n). In line with these results, the actual liver/body weight ratio was suppressed by Nox4 overexpression in AKO mice at 2 dpH (Fig. 8o, p). In addition, Nox4 overexpression increased the levels of ALT and AST in livers (Fig. 8q, r).

## Discussion

In this study, we provide in vitro and in vivo evidence demonstrating the functional importance of FoxO3 in regulating hepatocyte proliferation and liver regeneration in adult mice. We found that FoxO3 expression is downregulated in the early stage of partial hepatectomy (PH), which is accompanied by an increase in hepatocyte proliferation. Although the constitutive loss of FoxO3 in the liver developmentally restricts polyploidization and promotes hepatocyte proliferation, its loss in the adult liver alone further demonstrates that FoxO3 deficiency accelerating the speed of liver regeneration primarily depends on increased hepatocyte proliferation. Our data demonstrated that FoxO3 negatively regulates hepatocyte proliferation and liver regeneration by promoting *Nox4* expression and suppressing *Nr4a1* expression, thereby controlling functional regeneration of the liver.

Previous studies have demonstrated that FoxO3 mediates a variety of cellular processes including apoptosis, proliferation, and cell cycle progression^22^. Importantly, it has been demonstrated that FoxO3 plays critical roles in many liver diseases including liver damage^23^. In this study, we showed that FoxO3 expression decreased in the early stage of PH (Fig. 1), which was accompanied by an increased proliferation of hepatocytes (Fig. 3 and Supplementary Fig. 5). Furthermore, FoxO3 deficiency further enhanced the capacity for hepatocyte proliferation in vitro and in vivo (Figs. 3–5, Supplementary Figs. 3–5), indicating that FoxO3 negatively controls the proliferation of hepatocytes. In contrast to wild-type mice, *FoxO3* deficiency accelerated the speed of liver regeneration and restored the liver tissue size as early as 4 dpH (Figs. 3, 5, and Supplementary Fig. 5). However, massive upregulation of FoxO3 was observed at 28 and 56 dpH (Fig. 1b). Given that most of the original liver tissue is recovered 1–2 weeks post-PH in WT mice^1^, we speculate that the massive upregulation of FoxO3 in the later stage of PH may be responsible for preventing excessive regeneration, thereby ensuring that the regenerated liver is an appropriate functional size. Our data reveal that FoxO3 may restrict excessive liver regeneration by suppressing the proliferation of hepatocytes. This idea is further supported by a previous study that showed that activation of FoxO3 suppressed the proliferation of the liver hepatocellular cells lines HepG2 and MHCC-97H^24^. In addition to the hepatocytes tested in this study, FoxO3 is also critical for the regulation of the proliferation of hepatic stellate cells^25^ and can facilitate autophagy flux in Kupffer cells^26^. It is well known that EGF and HGF are considered liver regeneration initiators because they can facilitate liver cells entry into the cell cycle. These growth factors can excite the Ras-Raf-MEK-ERK and PI3K-PKB-mTOR cascades through their own receptors EGFR and MET (HGF receptor), thereby leading to hepatic cell division and repair^27^. We found that FoxO3-deficient hepatocytes are more sensitive to primary hepatocyte mitogens (Supplementary Fig. 6a, b). However, FoxO3 deficiency had no significant effects on the expression of EGFR and MET under our experimental conditions (Supplementary Fig. 6c, d). These findings reveal that the higher sensitivity of FoxO3-deficient hepatocytes to mitogens are EGFR- and MET-independent. The liver contains a mixture of hepatocytes with diploid or polyploid nuclear content in adult mammals. However, it has been demonstrated that diploid hepatocytes proliferate faster than polyploids, and that the polyploid state restricts hepatocyte proliferation and liver regeneration in mice^28^. Under our experimental conditions, constitutive loss of FoxO3 in developmental livers was found to restrict polyploidy and elevate the ratio of diploid hepatocytes (Fig. 4 and Supplementary Fig. 2), which may contribute to accelerated regeneration of the liver in AKO mice. It has been demonstrated that p53 deficiency greatly increases the number of polyploid hepatocytes during liver development and regeneration, suggesting that p53 is critical for limiting excessive hepatocyte polyploidization^29^. p53 can directly bind and activate the expression of FoxO3 in the adult mouse liver, whereas this binding and activation is disrupted during liver regeneration^18^. Consistent with this report, both the mRNA and protein levels of FoxO3 were indeed found to decrease in the early stage of liver regeneration in wild-type mice (Fig. 1). These data suggest that the restriction of hepatocyte polyploid by p53 is FoxO3-independent during liver regeneration.

Nicotinamide adenine dinucleotide phosphate (NADPH) oxidase (Nox) is a multicomponent enzyme complex that generates reactive oxygen species (ROS) in response to a wide range of stimuli^30^. Knockdown of Nox4 correlated with decreases in intracellular ROS levels and p21 activation and promoted expression of the cyclin D1 protein, thereby enhancing the proliferation of liver tumor and untransformed cells^31^. Consistently, our data reveal that *FoxO3*-knockout leads to decreased p27 and increased cyclin E1 expression during liver regeneration (Fig. 6i–l). Moreover, these previous data are in line with our results showing that *Nox4* silencing promotes hepatocyte proliferation in vitro as demonstrated by EdU nuclei incorporation and cell counting assay (Supplementary Fig. 23). Notably, both in vitro and in vivo data reveal that FoxO3 deficiency results in downregulation of *Nox4* (Fig. 8b–d), which was correlated with an increase in hepatocyte proliferation and liver regeneration (Fig. 3 and Supplementary Fig. 5). Importantly, overexpression of Nox4 in FoxO3-deficient livers attenuated the proliferation of hepatocytes and exacerbated liver damage during regeneration (Fig. 8). Consistent with our data, a previous study revealed that liver expression of *Nox4* decreases upon PH, which leads to increased proliferation of hepatocytes in the early stage of liver regeneration^31^. These data suggest that decreased *Nox4* expression is responsible for increased hepatocyte proliferation and liver regeneration in FoxO3-deficent mice. Additionally, our data indicate that the nuclear orphan receptor Nr4a1 is another potential target of FoxO3 during liver regeneration. *FoxO3* deficiency leads to upregulation of *Nr4a1* in the liver and hepatocytes. However, silencing *Nr4a1* greatly attenuates hepatocyte proliferation and liver regeneration in AKO mice (Fig. 7). These results suggest that the negative regulation of *Nr4a1* by FoxO3 is critical for hepatocyte proliferation and liver regeneration. Consistent with our results, previous studies have demonstrated that Nr4a1 promotes the proliferation of other cell types including colorectal cancer^32^ and vascular smooth muscle cells^33^. In line with our data, the functional importance of Nr4a1 in the liver has been reported in a previous study^34^.

In conclusion, our data show that FoxO3 expression is decreased in adult livers in the early stage of PH, which is correlated with an increase in hepatocyte proliferation. Using loss-of-function experiments in the constitutive Albumin-Cre driver line and AAV8-Tbg-Cre-injected FoxO3^fl/fl^ mice, we demonstrate that FoxO3-deletion promotes acute liver regeneration primarily by regulating the polyploidization and proliferation of hepatocytes, at least in part, through *Nox4* downregulation and *Nr4a1* upregulation. Given that a latest sequencing study revealed the role of mutant FoxO1 in driving hepatocyte proliferation through association in human liver disease^35^, our data implying the role of FoxO3 in liver regeneration in mice will further support the ideal that FoxO transcription factors might be important for the regulation of hepatocyte proliferation. A deeper understanding of the mechanism by which FoxO3 regulates hepatocyte proliferation and liver regeneration might lead to major therapeutic advances in the field of regenerative medicine. It should be noted that FoxO3 has pleiotropic roles and that its detailed function depends on different stimulation and/or different cell types. Thus, it is not suitable to directly translate the intrahepatic roles of FoxO3 reported in this study to other cell types.

## Methods

### Cell culture

NCTC1469, a liver cell line established in the 1957 from a neonatal mouse^36^, was obtained from the FuDan IBS Cell Center (FDCC, Shanghai, China). NCTC1469 cells were cultured in Dulbecco’s modified Eagle’s medium (DMEM) (Corning, USA) supplemented with 10% (v/v) Horse serum (Hyclone, Logan, UT, USA), 100 units/ml penicillin and 100 μg/ml streptomycin (Gibco, USA), at 37 °C in a 5% CO_2_ incubator. Culture medium was replaced every 2–3 days and cells were passaged when they reached 80% confluence. Cells were mycoplasma negative through treatment with LookOut® Mycoplasma Elimination Kit (Sigma-Aldrich). Gene silencing was achieved by transfecting predesigned siRNA duplexes (Supplementary Table 3) designed and synthesized by RiboBio (Guangzhou, China).

### Establishment of stable FoxO3 knockdown and overexpression cells using the NCTC1469 cell line

Stable knockdown of the FoxO3 gene in NCTC1469 cells was achieved by lentiviral based short-hairpin RNA delivery^37^. FoxO3 specific shRNA or negative control were cloned into pLOX-U6-Puro vectors for viral particle package in HEK 293 T cells. Infected NCTC1469 cells were selected by puromycin and expanded to form a stable sub-line. Knockdown efficiency was confirmed at both mRNA and protein levels. The shRNA sequences are as follows: FoxO3-shRNA: 5′-GGA ACT TCA CTG GTG CTA AGC-3′; negative shRNA (NC): 5′-ACT ACC GTT GTT ATA GGT G-3′. Stable cell line generated by FoxO3-shRNA and negative shRNA were named as shFoxO3 cells and shNC cells, respectively. To establish FoxO3 overexpression stable cells, NCTC1469 cells were transfected by pLOX-FoxO3 lentiviruses^19^ and screened by puromycin and expanded to form a stable sub-line.

### In vitro cell proliferation assay

For cell proliferation assay, NCTC1469 cells were incubated for 48 h in 24 well plates with different treatments. DNA synthesis was then analyzed by 5-Ethynyl-2'-deoxyuridine (EdU) labeling, using Cell-Light™ EdU Apollo®567 In Vitro Imaging Kit (RiboBio, Guangzhou, China) according to the manufacturer’s instructions. At least seven images were randomly taken per well using a Zeiss LSM 700 laser confocal microscope (Carl Zeiss). The population of EdU^+^ cells was determined by counting at least 500 cells per well. The EdU^+^ cells were quantified as the percentage of total cells. In addition, cell proliferation was also analyzed by evaluating cell count using the Enhanced Cell Counting Kit-8 (CCK-8, Beyotime Biotechnology, China) according to the manufacturer’s instructions.

### Overexpression vector construction

Total RNA was isolated using RNeasy kit (Qiagen, Valencia, CA, USA) from NCTC1469 cells according to the manufacture’s instruction. The cDNA was transcribed from total RNA using SuperScript III Reverse Transcriptase (Roche, USA). The coding sequence (CDS) of *Nox4* (NM_015760.5) was amplified by the KOD-Plus-Neo Kit (Toyobo, Japan) and cloned into the pcDNA3.1 expression vector to construct the pcDNA-Nox4 plasmid. Constructed overexpression vector pcDNA-Nox4 and control plasmid pcDNA3.1 (pcDNA) were transfected into the FoxO3-overexpressing NCTC1469 cells to explore the effect of target gene on FoxO3-mediated proliferation and apoptosis.

### Animals

FoxO3-LoxP-targeted (*FoxO3*^*fl/fl*^) mice (C57BL/6 background) were created by Cyagen Biosciences (Suzhou, China)^19^. The exon 3 region of the FoxO3 gene was flanked by LoxP sites and deleted upon Cre-mediated recombination (Fig. 2a). The *Albumin* (*Alb*)-*Cre* transgenic line was obtained from the Jackson Laboratory (stock number 003574). *Alb-Cre* mice were genotyped using oIMR5374 (GAA GCA GAA GCT TAG GAA GAT GG) and 20240 (TTG GCC CCT TAC CAT AAC TG) primers and identified by a 390 bp PCR product. For *Alb-Cre* mice, *Alb* promoter drive Cre recombinase expression primarily in hepatocytes with variable expression in cholangiocytes depending on the floxed gene^38,39^. *FoxO3*^*fl/fl*^ mice were intercrossed with *Alb-Cre* mice to generate *FoxO3*^*fl/+*^*::Alb-Cre* (*FoxO3*^*+/-*^, heterozygote). Heterozygotes were then crossed with *FoxO3*^*fl/fl*^ mice to generate *FoxO3*^*fl/fl*^*::Alb-Cre* mice (*FoxO3*^*-/-*^, hereafter termed AKO) with constitutive loss of FoxO3 in hepatocytes. The *FoxO3*^*fl/fl*^ mice obtained from the same breeding were used as control mice (Con). Age-matched *FoxO3*^*fl/fl*^ mice were used as control (Con). To induce conditional knockout of *FoxO3* in adult liver alone, adeno-associated virus serotype 8 (AAV8) was constructed to specifically express Cre recombinase in liver. To confirm the recombination efficiency of Cre, AAV8-Tbg-Cre (AAV-Cre) virus was intravenously injected into R26-CAG-LSL-EGFP mice (Shanghai Model Organisms Center, Shanghai, China) with different concentrations. Two weeks later, the percentages of EGFP^+^ liver cells were then checked to evaluate Cre recombination efficiency. We found that AAV-Cre adeno-associated virus with the concentration of 5 × 10^11^ gc/mouse has a highest efficiency (Supplementary Fig. 11). Therefore, AAV-Cre or negative control (AAV-NC) adeno-associated virus was injected intravenously at a concentration of 5 × 10^11^ gc/mouse in *FoxO3*^*fl/fl*^ mice 2 weeks prior to partial hepatectomy (PH). For knockdown of Nr4a1 in adult AKO mice, AAV8-mediate shRNA targeting for Nr4a1 (AAV-shNr4a1) or negative control (AAV-shNC) adeno-associated virus was injected intravenously at a concentration of 5 × 10^11^ gc/mouse in AKO mice 2 weeks prior to partial hepatectomy (PH). To overexpress Nox4 in AKO mice, AAV8-Tbg-Nox4 (AAV-Nox4) or negative control (AAV-NC) adenovirus was injected intravenously at a concentration of 5 × 10^11^ gc/mouse in FoxO3-deficient mice 2 weeks prior to liver injury. For experiments using adult animals, male mice were used in this study. All animal protocols and procedures were approved by the Institutional Animal Care and Use Committee of Jinan University. Data and methods are reported here in accordance with ARRIVE guidelines^40^.

### Liver injury models

Male animals aged 8 ~ 9 weeks were kept on a 12 h-day/night cycle with free food/water access, and were used for partial hepatectomy (PH) experiments. During anesthesia induced by isoflurane, mice were subjected to 70% PH or 30% PH surgery. The surgery was performed between 8 and 12 am to remove the median and left lobes and the mortality was <5%. The weight of regenerated livers as well as the whole body was measured at 0–7 days post-PH (dpH). The ratio of wet weight of the remaining liver after PH over the weight of the whole animal was taken as the liver to body weight ratio. The 0 dpH denotes the time point in quiescent animal before PH. To induce another classic liver injury model, male adult mice were injected with CCl_4_ (0.6 mL/kg, intraperitoneally) diluted in olive oil three times a week (every 48 h) for 4 weeks and euthanized 48 h after the last injection as previously reported^41^.

### Ploidy analysis and growth assay of primary hepatocytes

Mouse livers were flushed via the portal vein using perfusion buffer containing Collagenase Type IV, followed by primary hepatocyte isolation and purification. Isolated and purified hepatocytes were fixed at 4 with 70% ethanol overnight at 4 °C, washed three time with PBS, and incubated with propidium iodide (PI, 50 μg/ml, Sigma)/RNAse A (ribonuclease, 1 mg/ml, Sigma) solution for 3 h at room temperature, followed by flow cytometry analysis with a FACS Aria cytometer (Becton Dickinson) to determine DNA content and ploidy distribution using ModFit LT 5.0 software (Verity Software House). Growth assay of primary hepatocytes were performed as previously report^21^. In brief, isolated hepatocytes were cultured in mouse hepatocyte growth medium (MHGM) with or without mitogens including EGF and HGF (Gibco, 40 ng/ml for each) for 4 days as described previously. DNA synthesis were then analyzed by 5-Ethynyl-2'-deoxyuridine (EdU) labeling, using Cell-Light™ EdU Apollo®567 In Vitro Imaging Kit (RiboBio, Guangzhou, China) according to the manufacturer’s instructions.

### Histology

Liver tissues were fixed in 4% formaldehyde and dehydrated in a series of ethanol. The fixed liver was embedded in paraffin and sectioned to 5 μm thickness. Sections were subjected to hematoxylin and eosin (H&E) staining for morphological and mitotic analysis. Paraffin-embedded liver sections were deparaffinized in xylene, followed by rehydration through a graded ethanol series and staining with hematoxylin and eosin stain (H&E). Mitotic activity in liver was examined and quantified as previously described^2^. In brief, mitotically active areas were first screened under lower magnification. For quantification, total mitotic counts in five high-magnification fields in the most mitotically active areas were considered for each mouse using a light microscope coupled with a digital image acquisition system.

### Immunofluorescence and immunohistochemistry staining

Liver sections were subjected to immunofluorescence staining to analyze the expression of cell proliferating markers using a confocal fluorescence microscope^42^. In brief, livers were embedded in paraffin and cut in 5 μm sections, followed by deparaffinization and rehydration. Antigen retrieval were routinely performed using Citrate Antigen Retrieval Solution (Beyotime Biotechnology, P0081) according to the manufacturer’s instructions. Sections were permeabilized with 0.5% Triton X-100/PBS and then blocked with 5% goat serum (Jackson ImmunoResearch Laboratories, USA) for 1 h at room temperature, and incubated with appropriate primary antibodies overnight at 4 °C. After washing with PBS, sections were incubated with corresponding secondary antibodies conjugated to fluorescence for 1 h at room temperature, followed by counterstaining with DAPI (Sigma). Secondary antibodies used are following: Alexa Fluor 488 donkey anti-rabbit IgG (Abcam, 1:500), Alexa Fluor 405 donkey anti-goat IgG (Abcam, 1:200), and Cy3-conjugated Affinipure Goat anti-rabbit IgG (Proteintech, 1:100). For immunohistochemistry staining, sections were stained with primary antibody overnight at 4 °C. SignalStain® Boost IHC Detection Reagents (HRP, CST) and SignalStain® DAB Substrate Kit (CST) were used for amplification and development, respectively. The detailed information of primary antibodies used in this study can be found in the Supplementary Table 4. For immunostaining in liver tissues, species isotype (Santa Cruz) was used as negative control to confirm the specificity of primary antibodies. Images were captured by laser-scanning confocal microscope (LSM 700, Zeiss) and analyzed by ZEN 2012 software (Zeiss). Given that FoxO3 was predominantly expressed in nuclei of liver cells (Fig. 1a, b), FoxO3 fluorescence intensity in nuclei were normalized to nuclei size (mean fluorescence intensity, MFI) over total slides using ZEN 2012 software (Zeiss) to evaluate the total expression levels of FoxO3 protein. For the quantification of cell proliferation, the proliferation of hepatocytes (HNF4α^+^ cells) was quantified by the percentage of proliferating hepatocytes (Ki67^+^ HNF4α^+^ cells, or pH3^+^ HNF4α^+^ cells) relative to total hepatocytes (HNF4α^+^ cells). For some experiments, the proliferation of non-hepatocytes (HNF4α^−^ cells) was quantified by the percentage of proliferating non-hepatocytes (Ki67^+^ HNF4α^−^ cells, or pH3^+^ HNF4α^−^ cells) relative to total liver cells (DAPI^+^ cells).

### Hepatocyte size estimation

Hepatocyte size was evaluated by β-catenin staining. In brief, following deparaffinized, rehydrated, slides were then incubated with anti-β-catenin (BD, 1:500) overnight at 4 °C, followed by a further incubation at room temperature for 1 h with goat anti-rabbit IgG H&L (Alexa Fluor 488) preadsorbed secondary antibody. Nuclear DNA was labeled in blue with DAPI. To quantify the cell size, 5 independent livers per group (at least 300 cells) were captured with laser-scanning confocal microscope (LSM 700, Zeiss). ZEN 2012 lite software (Zeiss) was used to quantify the relative size of each cell. Moreover, the relative size of primary hepatocytes was also evaluated by the forward scatter (FSC) intensity in flow cytometry.

### EdU labeling in vivo

For EdU labeling experiments, mice were intraperitoneally injected with 100 μl of a 1 mg/ml solution of EdU (RiboBio, Guangzhou, China) dissolved in PBS. Livers were embedded in Tissue-Tek optimal cutting temperature compound (OCT) (Sakura, USA) for frozen section (4 μm). Sections were rinsed three times in PBS and fixed in 4% parapormaldehyde for 30 min. After rinsing three times again, citrate antigen retrieval was performed as described above. Sections were then incubated with 2 mg/mL glycine solution for 10 min, permeabilized with 0.5% Triton X-100 in PBS for 10 min, and then rinsed with PBS once for 5 min. This was followed by incubation with Apollo®576 staining solution (1×) at room temperature for 30 min. Sections were washed three times in PBS, stained with DAPI for 10 min to label nuclei, and mounted in Antifade Mounting Medium. Images were captured by a laser-scanning confocal microscope (LSM 700, Zeiss) and analyzed by ZEN 2012 software (Zeiss).

To analyze hepatocyte proliferation at the indicated time points, EdU was injected 2 h prior to liver collection. For EdU pulse-chase experiments, EdU was multiply injected at indicted time points to label all proliferating hepatocytes during the whole period of liver regeneration. The last injection was performed 2 h prior to liver collection. Sham-operated mice underwent the same procedure without the liver resection. For the quantification of cell proliferation, the proliferation of hepatocytes (HNF4α^+^ cells) was quantified by the percentage of proliferating hepatocytes (EdU^+^ HNF4α^+^ cells) relative to total hepatocytes (HNF4α^+^ cells). But the proliferation of non-hepatocytes (HNF4α^-^ cells) was quantified by the percentage of proliferating non-hepatocytes (EdU^+^ HNF4α^-^ cells) relative to total liver cells (DAPI^+^ cells).

### Serum and tissue enzymatic activity

Blood was collected by shearing the right atrium and was allowed to coagulate for 2 h on ice. Serum was the isolated as the supernatant fraction after centrifugation at 2000 rpm for 5 min. Hepatic function was analyzed using serum ALT and AST activities, which are markers of injury. Serum ALT and AST activities were determined using the Hitachi 7600 automatic biochemical analyzer (Japan) and were expressed as units per liter (U/L). In addition, enzyme activities in liver tissue were determined using commercial kits produced by Jiancheng Institute of Biotechnology (Nanjing, China). Tissue enzyme activities were normalized to total protein weight and were expressed as units per gram protein (U/g).

### RNA extraction, quantification, and sequencing

For total RNA isolation, liver tissues were extracted in sham and injured mice at 4 days post-PH (dpH), respectively. Three mice per group were used for RNA-sequencing analysis. RNA preparation, library construction and sequencing on GBISEQ-500 platform was performed. After filtering the reads with low quality, clean reads were then obtained and mapped to the reference genome of mouse (GRCm38.p6) with the HISAT^43^. Gene expression level was quantified by a software package called RSEM^44^ and expressed as fragments per kilobase of exon per million fragments mapped (FPKM). Differential expressed (DE) genes were detected using NOISeq method^45^ with Probability ≥ 0.8 and fold change (FC) of FPKM ≥ 2. Only those genes were considered for the differential expression analysis, which displayed FPKM ≥ 1 in either of the two samples under comparison. GO analysis was performed using online tool DAVID 6.8 (https://david.ncifcrf.gov/summary.jsp), and terms with *p-*value ≤ 0.05 were included. Differentially expressed gene heat maps were clustered by hierarchical clustering using cluster software^46^. RNA sequencing data have been deposited in NCBI Sequence Read Archive (SRA) under the accession code SRP224045.

### RNA extraction and quantitative Real-Time PCR (qPCR)

Total RNA was isolated using RNeasy kit (Qiagen, Valencia, CA, USA) from cells or liver tissue according to the protocol of the manufacturer, respectively. Reverse transcription to cDNA was performed with 30 ng of total RNA, random primers, and SuperScript III Reverse Transcriptase (Roche, USA). The qPCR was performed using a Light Cycler 480 SYBR Green I Master (Roche, USA) and the MiniOpticon qPCR System (Bio-Rad, CA, USA). After denaturation for 10 min at 95 °C, the reactions were subjected to 45 cycles of 95 °C for 30 s, 60 °C for 30 s, and 72 °C for 30 s. GAPDH was used as the internal standard control to normalize gene expression using the 2^−△△Ct^ method. The sequences of the qPCR primers were listed in Supplementary Table 5.

### Protein extracts and western blotting

Tissues or cells were lysed in RIPA buffer (Beyotime Biotechnology) containing protease inhibitors (Sigma) for SDS-PAGE. Protein concentrations were determined using Bio-Rad Protein Assay (Bio-Rad Laboratories). 30 μg of protein were separated by SDA-PAGE, proteins were transferred onto PVDF membranes (Millipore), then blocked in 5% nonfat milk/TBS-Tween 20 and incubated with primary antibodies (dilution in TBST) overnight at 4°C. Membranes were then washed and incubated with corresponding second antibodies for 1 h at room temperature. Bands were detected by chemiluminescence reagents (ThermoFisher Scientific). Primary antibodies can be found in the Supplementary Table 4. Secondary antibodies used are following: goat-anti-mouse horseradish peroxidase (HRP)-conjugated antibody (CST, 1:3000) and goat-anti-rabbit horseradish peroxidase (HRP)-conjugated antibody (CST, 1:2000). Regarding the visualization of housekeeping protein (β-actin), the partial membrane was cropped from the same one as the target proteins of interest when their molecular weights are greatly different from each other. Otherwise, two members with same loading dose of total protein were simultaneously performed to visualize the target protein and β-actin, respectively. Given that stripping and re-probing may result in errors between different bands, stripping buffer was not used in this study. Chemiluminescent signals were quantitated with Image-Pro Plus version 6.0 software (Media Cybernetics, MD, USA). The expression level of target protein was set as 100% in control group. Relative expression levels of target protein in experimental groups were expressed as the percentage of control group. All blots derive from the same experiment and were processed in parallel. The uncropped blots are listed in the Supplementary Figs. 24 and 25.

### Luciferase reporter assay

The promoter sequences (−2000 bp to −1 bp, upstream of TSS) of the mouse *Nox4* (Gene ID: 50490) and *Nr4a1* (Gene ID: 15370) genes were analyzed by JASPAR 2018 online software (http://jaspar.genereg.net/)^47^ to determine potential FoxO3 binding sites (Supplementary Tables 1 and 2). The predicted binding site with highest score for target gene promoters were further analyzed by luciferase reporter assay, to evaluate the regulation effects of FoxO3 on the expression of mouse *Nox4* and *Nr4a1* genes. The pCMV-Gaussia-Dura Luc and pTK-Red Firefly Luc plasmids from ThermoFisher were used to construct the dual-luciferase reporter plasmid (pGL-RF) (Supplementary Figs. 19b and 22b), which contained both a Gaussia luciferase (GL) and a red firefly luciferase (RF). The predicted binding sites with highest score for target gene promoters were further analyzed by pGL-RF luciferase dual reporter assay, to evaluate the in vitro binding and regulating effects of FoxO3 on *Nox4* and *Nr4a1* genes. Briefly, promoter regions of target genes comprising predicted FoxO3 binding sites were amplified and cloned into the pGL-RF plasmid. NCTC1469 cells were co-transfected with dual reporter plasmids and pcDNA-FoxO3 plasmid (50 ng for each plasmid) using LipoFiter Liposomal Transfection Reagent (Hanbio Biotechnology). Two days after transfection, luciferase reporter assay was carried out using the Pierce™ Gaussia-Firefly Luciferase Dual Assay Kit (ThermoFisher Scientific) according to the manufacturer’s protocol. Luciferase activity was measured using a BioTek Synergy^TM^ 4 multimode microplate reader (BioTek Instruments). The relative activity of the *Gaussia* luciferase (GL) was normalized by the activity of red firefly luciferase (RL) and was expressed as fold change of control group. To further verify the potential binding sites, mutant luciferase reporter plasmids were generated by KOD-Plus-Mutagenesis Kit (Toyobo, Osaka, Japan), according to the manufacturer’s protocol.

### Chromatin immunoprecipitation (ChIP) assay

ChIP assays were performed to evaluate the in vivo binding of FoxO3 to its consensus sequence in mouse *Nr4a1* and *Nox4* promoters. The assays were performed in mouse liver tissue using the SimpleChIP® Plus Enzymatic Chromatin IP Kit (CST, #9004) according to the manufacturer’s instructions. FoxO3 antibody (CST, #2497) was used to immunoprecipitation assay. Normal goat IgG (CST, #2729) was used as a control as previously described. The DNA isolated from input chromatin fragments and from the precipitated chromatin fragments by anti-FoxO3 antibody or control IgG was subjected to PCR using primers flanking the consensus FoxO3 binding sites on *Nox4* promoter. PCR products were determined on a 1.5% agarose gel. Relative binding ability of FoxO3 was expressed as the DNA signals relative to input. ChIP-PCR primers used in this study as follows: Nr4a1-ChIP-F: 5'-GGC CTC ACT TTT TCC ACC TAG T-3' and Nr4a1-ChIP-R: 5'-CCA GGG TAG GGT TGC TGT TTC-3'; Nox4-ChIP-F: 5'-TTG ACT TTG CAA TTA GCA GTA-T-3' and Nox4-ChIP-R: 5'-AGT CAG AAG CCC AAG TCT TCC T-3'.

### Statistical analysis

All statistics were calculated using GraphPad Prism 8 Software. Among three or more groups, statistical analysis was performed using one-way or two-way ANOVA with Dunnett’s multiple comparisons post hoc tests. Comparisons between two groups were analyzed using unpaired and 2-tailed Student’s *t*-test. All data are presented as the mean ± SEM. A *p*-value < 0.05 was considered statistically significant. In this study, statistical analysis was in accordance with homogeneity of variance and normality of residuals. Homogeneity of variance was evaluated by the *F*-test with a *p*-value of >0.05. Normality of residuals was evaluated by D'Agostino–Pearson test and/or Shapiro–Wilk test with a *p*-value of >0.05.

### Reporting summary

Further information on research design is available in the Nature Research Reporting Summary linked to this article.

## Supplementary information

Supplemental Material

REPORTING SUMMARY

## Data Availability

The data that support the findings of this study are available from the corresponding author upon reasonable request. RNA-sequencing data have been deposited in NCBI Sequence Read Archive (SRA) under the accession code SRP224045.
