# Supplementary material for: FoxO3 restricts liver regeneration by suppressing the proliferation of hepatocytes
Source: NPJ Regen Med. 2022 Jun 24;7:33. doi: 10.1038/s41536-022-00227-6 (PMC9232540; doi:10.1038/s41536-022-00227-6)
Supplement: Supplementary file 1 — Supplemental Material [file 41536_2022_227_MOESM1_ESM.pdf]

Supplementary Material

FoxO3 restricts liver regeneration by suppressing the proliferation of hepatocytes

Chi-Qian Liang, Deng-Cheng Zhou, Wen-Tao Peng, Wu-Yun Chen, Hai-Yan Wu, Yi-Min Zhou, Wei-Li Gu, Kyu-Sang Park, Hui Zhao, Long-Quan Pi, Li Zheng, Shan-Shan Feng, Dong-Qing Cai, Xu-Feng Qi

Supporting figures and figure legends

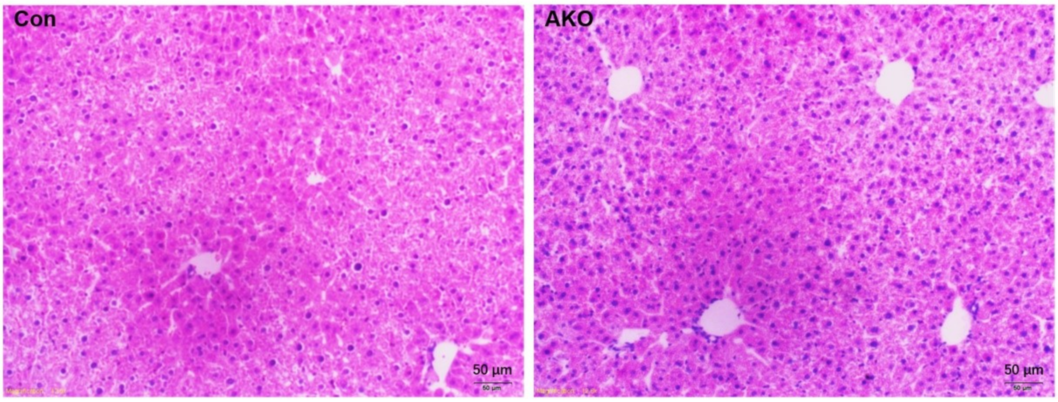

Supplementary Figure 1. Histology of livers from knockout and control mice at 8 weeks of age (scale bar=50 μm).

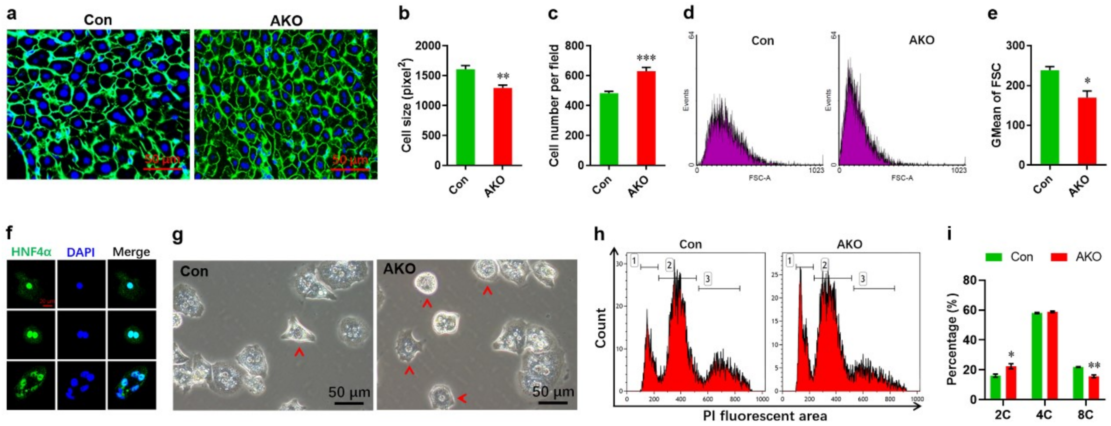

Supplementary Figure 2. Effects of the constitutive loss of FoxO3 on diploidy of hepatocytes. (a-c) The cell size of hepatocytes was analyzed by β-catenin staining. Representative images (a) and quantification (b) of the cell size are shown (*n*=5 mice). (c) Quantification of cell numbers in control and knockout livers (*n*=10 mice). (d and e) The cell size of primary hepatocytes isolated from Con and AKO mice were evaluated by the forward scatter (FSC) intensity in flow cytometry. Representative images (d) and quantification (e) are shown (*n*=3 mice). (f) Representative images of primary hepatocytes with different nuclei were identified by HNF4α (green) and DAPI (blue) double staining. (g) Representative images of primary hepatocytes isolated from livers in Con and AKO mice. Arrowhead denotes mononuclear hepatocytes. (h and i) The ploidy of primary hepatocytes isolated from Con and AKO mice were analyzed using flow cytometry. Representative images (h) and quantification (i) are shown (*n*=3 mice). All data are presented as the mean ± SEM. *$p<0.05$, **$p<0.01$, ***$p<0.001$ versus controls (Student's *t*-test for b, c, and e; two-way ANOVA test for i).

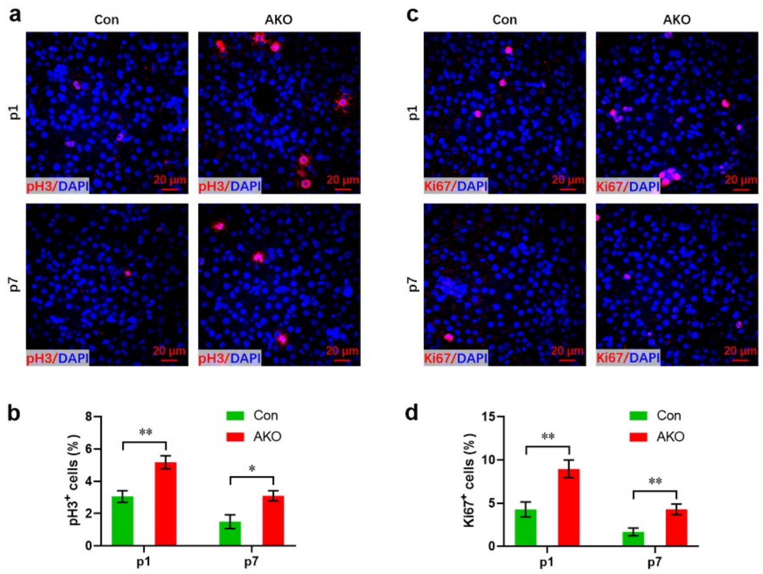

Supplementary Figure 3. FoxO3 mutation promotes the proliferation of liver cells in postnatal mice. Livers from neonatal mice at postnatal day 1 (p1) and p7 were submitted to immunofluorescent staining to determine cell proliferation. (a and b) Representative images (a) and quantification (b) of $pH3^+$ liver cells in neonatal mice at p1 and p7. Data are presented as the mean ± SEM (n=5 mice per group), *p<0.05, **p<0.01 versus Con mice. (c and d) Representative images (c) and quantification (d) of $Ki67^+$ liver cells in neonatal mice at p1 and p7. Data are presented as the mean ± SEM (n=5 mice per group). **p<0.01 versus Con mice (two-way ANOVA test).

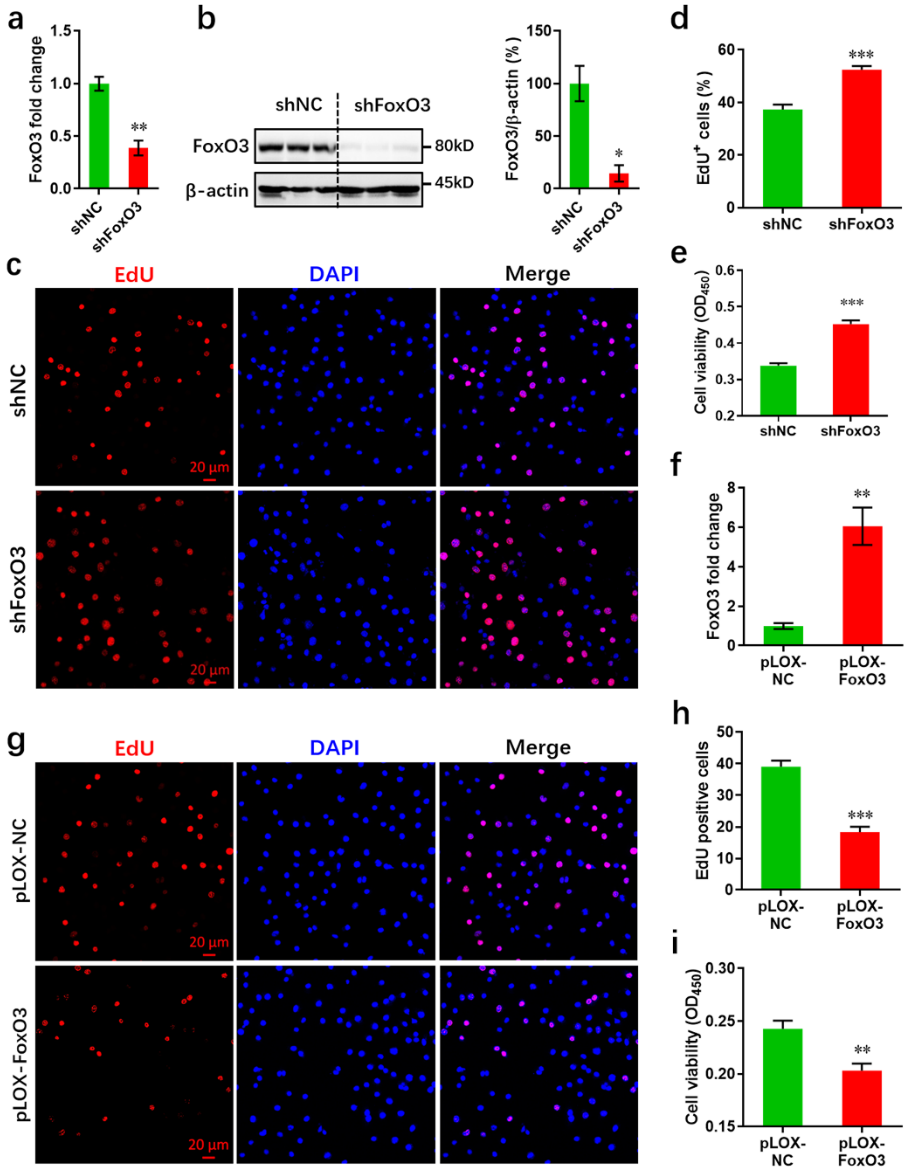

Supplementary Figure 4. FoxO3 plays an important role in the regulation of liver cell proliferation *in vitro*. (a-e) NCTC1469 cells are used to establish a stable FoxO3 knockdown cell line through lentiviral-mediated expression of an shRNA directed against FoxO3 (shFoxO3). The knockdown efficiency in mRNA (a) and protein (b) levels are verified by qPCR and western blot, respectively. Data are presented as the mean ± SEM (n=3 per group), **p<0.01 versus control. Representative images of EdU labeling (c) and quantification of EdU$^+$ cells (d) are shown (n=8~9 per group). Data are presented as the mean ± SEM. ***p<0.001. (e) Cell viability was examined using CCK8 Cell Counting Kit (E) (n=4 per group). Data are presented as the mean ± SEM. **p<0.01. (f-i) NCTC1469 cells are used to establish a stable FoxO3 overexpression cell line through lentiviral-mediated expression of FoxO3. The overexpression of FoxO3 is verified by qPCR (f). Data are presented as the mean ± SEM (n=3 per group), **p<0.01 versus control. Representative images of EdU labeling (g) and quantification of EdU$^+$ cells (h) are shown (n=6~8 per group). Data are presented as the mean ± SEM. ***p<0.001. (i) Cell viability was examined using CCK8 Cell Counting Kit (n=6 per group). Data are presented as the mean ± SEM. **p<0.01 (Student's t-test).

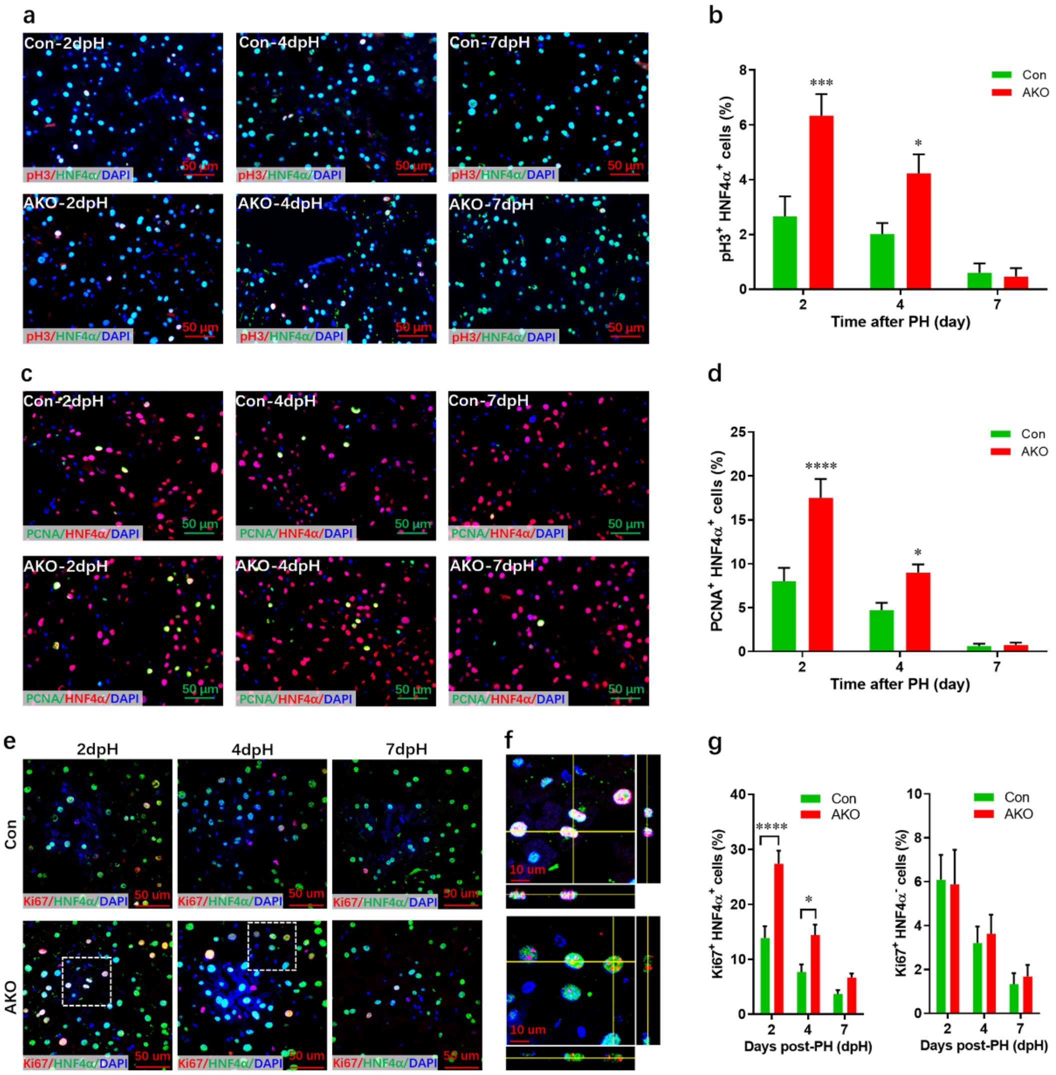

Supplementary Figure 5. Effects of constitutive loss of FoxO3 on hepatocyte proliferation during liver regeneration in adult mice. (a and b) Representative images (a) and quantification (b) of $pH3^{+}$ $HNF4\alpha^{+}$ cells in control and AKO mice post-PH (*n*=12 mice per group). (c and d) Representative images (c) and quantification (d) of $PCNA^{+}$ $HNF4\alpha^{+}$ cells in control and AKO mice post-PH (*n*=12 mice per group). (e) Representative images of $Ki67^{+}$ $HNF4\alpha^{+}$ cells in control and AKO mice post-PH. (f) Representative Z-stack confocal images of $Ki67^{+}$ $HNF4\alpha^{+}$ cells in AKO mice at 2 (upper) and 4 (lower) dpH (areas denoted by dotted line in e). (g) Quantification of $Ki67^{+}$ $HNF4\alpha^{+}$ (left) and $Ki67^{+}$ $HNF4\alpha^{-}$ (right) cells in control and AKO mice post-PH (*n*=10 mice per group). All data are presented as the mean ± SEM. *$p<0.05$, ***$p<0.001$, ****$p<0.0001$ versus control mice (two-way ANOVA test).

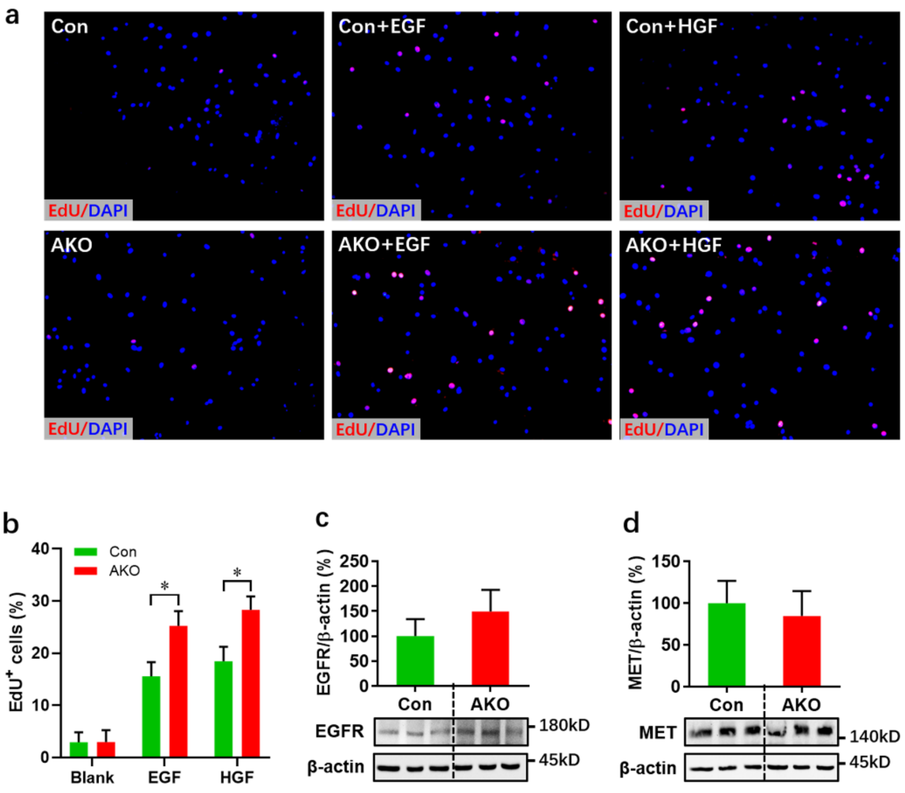

Supplementary Figure 6. Response of FoxO3-deficient hepatocytes to primary mitogens. Primary hepatocyte isolated from control and AKO adult mice were stimulated with EGF (40 ng/ml) and HGF (40 ng/ml) for 4 days in mouse hepatocyte growth medium (MHGM), followed by EdU incorporation assay. Representative images of EdU labeling (a) and quantification of EdU^+ cells (b) are shown (n=4 per group). (c and d) Representative western blot images (lower panel) and quantification (upper panel) of EGFR (c) and MET (d) expression in primary hepatocytes isolated from control and AKO adult mice (n=3 mice). Data are presented as the mean ± SEM. *p<0.05 (two-way ANOVA test).

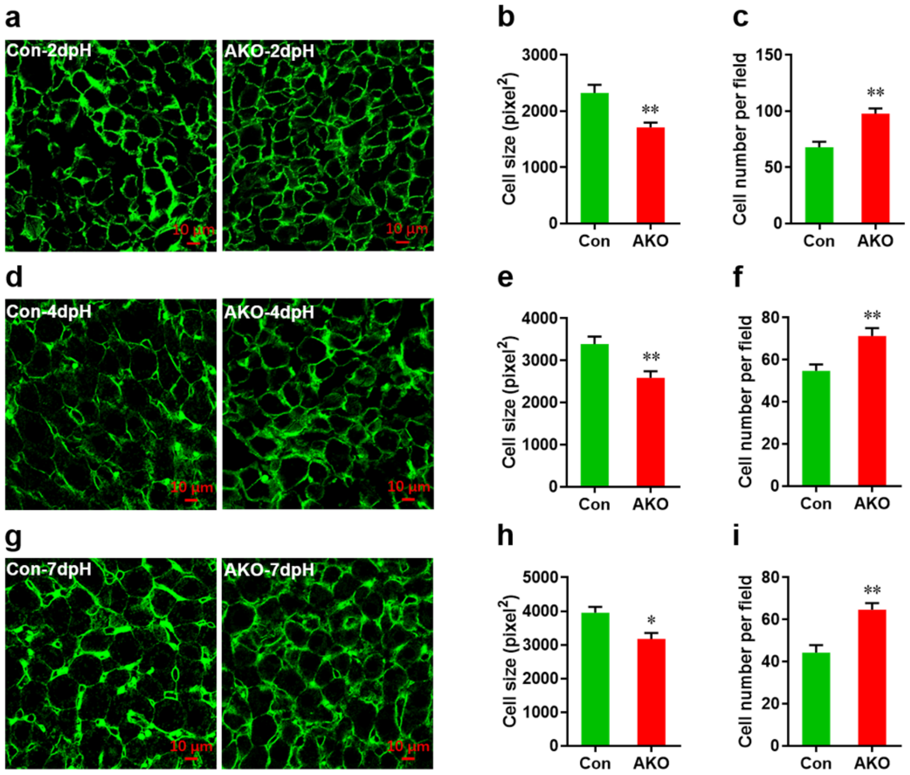

Supplementary Figure 7. Increased cell number of hepatocytes in AKO mice during liver regeneration. (a) Representative images of β-catenin staining in livers from control and AKO mice at 2 dpH. (b and c) Quantification of hepatocyte size (b) and density (c) in livers from control and AKO mice at 2 dpH. Data are presented as the mean ± SEM (*n*=5 mice per group). ***p*<0.01 versus control mice. (d) Representative images of β-catenin staining in livers from control and AKO mice at 4 dpH. (e and f) Quantification of hepatocyte size (e) and density (f) in livers from control and AKO mice at 4 dpH. Data are presented as the mean ± SEM (*n*=5 mice per group). ***p*<0.01 versus control mice. (g) Representative images of β-catenin staining in livers from control and AKO mice at 7 dpH. (h and i) Quantification of hepatocyte size (h) and density (i) in livers from control and AKO mice at 7 dpH. Data are presented as the mean ± SEM (*n*=5 mice per group). **p*<0.05, ***p*<0.01 versus control mice (Student's *t*-test).

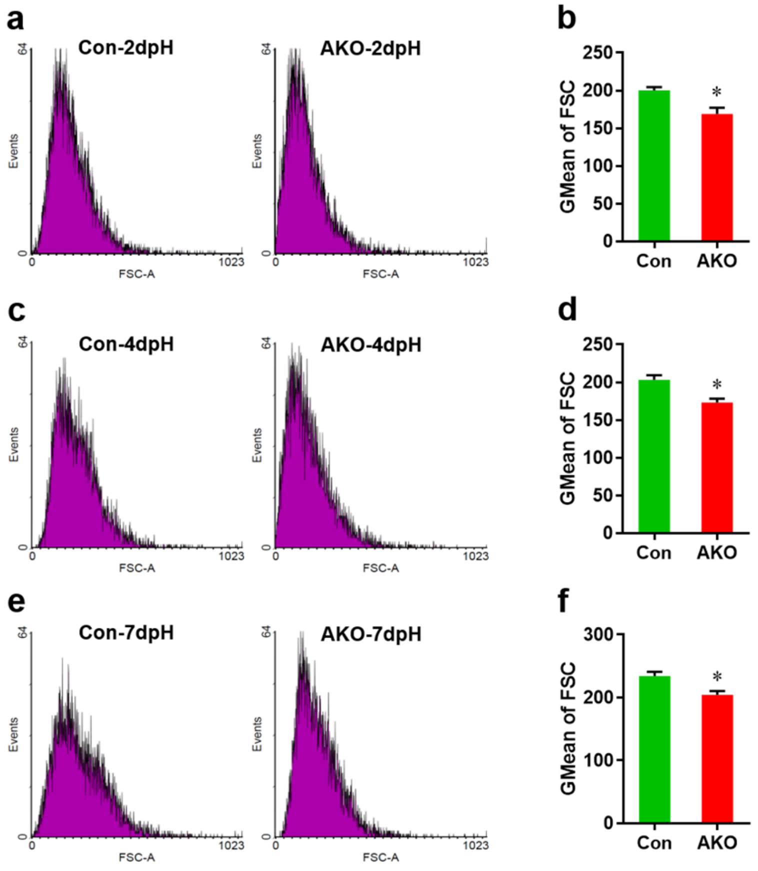

Supplementary Figure 8. The relative size of primary hepatocytes isolated from control and AKO mice during liver regeneration. The cell size of primary hepatocytes isolated from control and AKO mice were evaluated by the forward scatter (FSC) intensity in flow cytometry. Representative images (a, c, e) and quantification (b, d, f) are shown (*n*=3 mice). Data are presented as the mean ± SEM (*n*=3 mice per group). *$p<0.05$ versus control mice (Student's *t*-test).

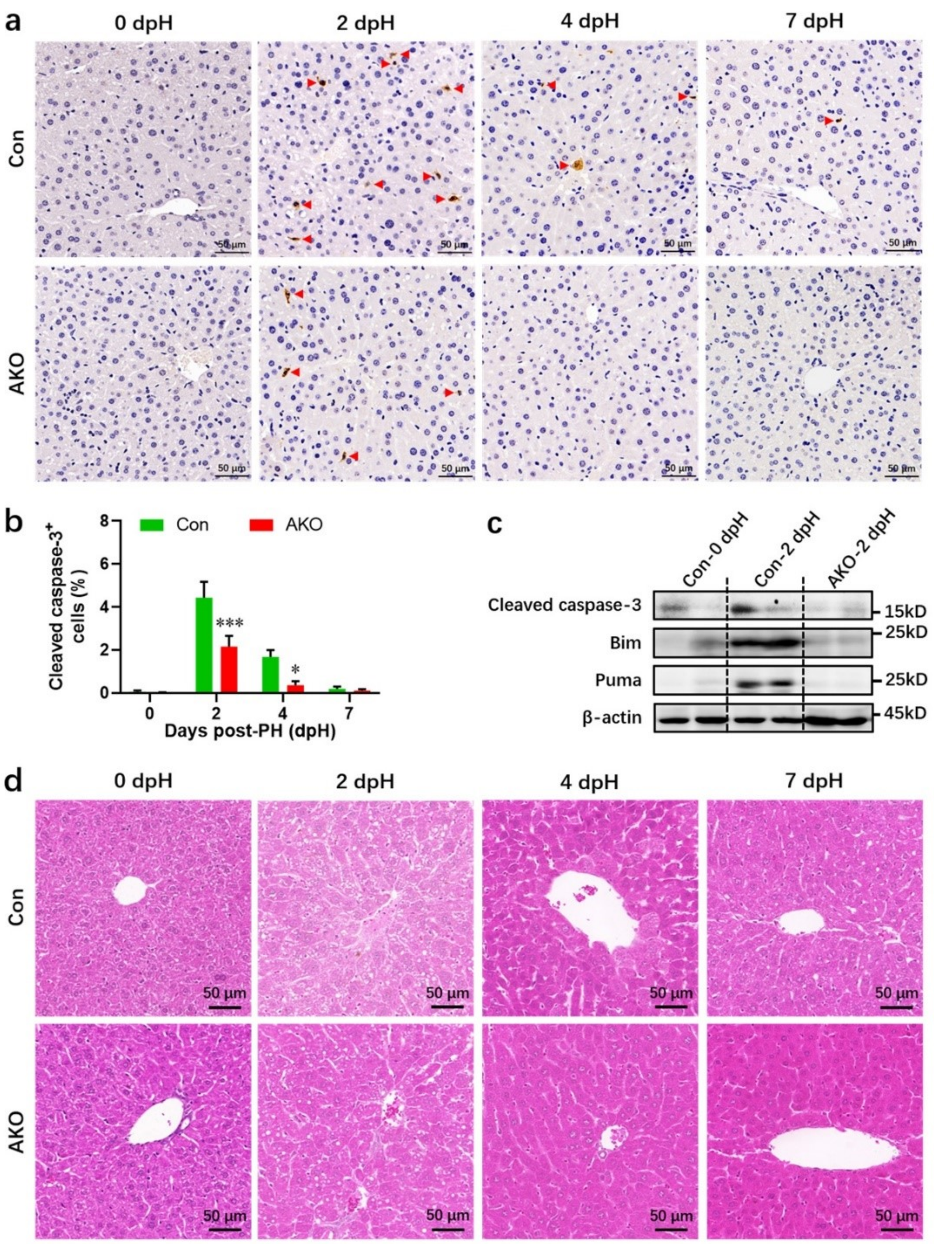

Supplementary Figure 9. FoxO3 deficiency reduces hepatic apoptosis in mice during liver regeneration. Liver tissues from mice at indicated time points (0 to 7 dpH) during regeneration were submitted to western blotting analysis using anti-cleaved caspase-3 antibody. (a and b) Representative images of immunohistochemical staining (a) and quantification (b) of claeved-caspase-3^{+} liver cells (brown) in control and AKO mice are shown. Data are presented as the mean ± SEM (n=5 mice per group). *p<0.05 versus control mice (two-way ANOVA test). (c) Activation of cleaved-caspase-3 and proapoptotic proteins (Bim and Pum) in control (0 and 2 dpH) and AKO (2 dpH) livers were analyzed using western blotting. (d) Histopathological analysis of the liver sections of control and AKO mice during regeneration.

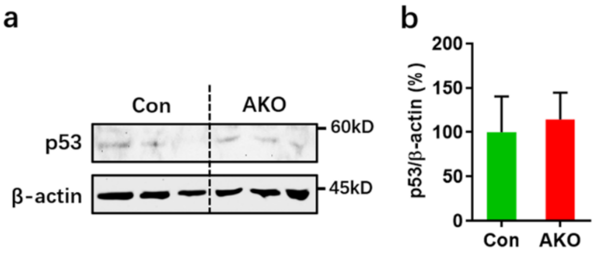

Supplementary Figure 10. Effects of FoxO3 on the expression of p53 in livers during regeneration. Liver from AKO and control mice were subjected to Western blotting assy. Representative images of (a) and relative quantification (b) of p53 protein levels in livers isolated from control and AKO mice at 2 dpH. Data are presented as the mean ± SEM (*n*= 3 mice per group).

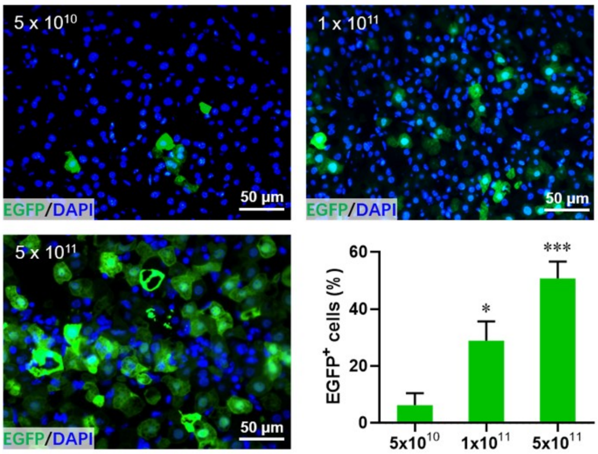

Supplementary Figure 11. Efficiency of AAV-Tbg-Cre virus-induced recombination efficiency in livers. Adult R26-CAG-LSL-EGFP mice were injected with AAV-Tbg-Cre virus with different concentrations (5×10^{10} ~ 5×10^{11} gc/mouse)2 weeks prior to reporter gene analysis. Liver tissues was then isolated for assessment of EGFP expression using immunofluorescent staining. The percentage of $EGFP^{+}$ liver cells were quantified to evaluate the recombination efficiency of AAV-Tbg-Cre. Data are presented as the mean ± SEM (*n*=3 mice per group). *$p<0.05$, ***$p<0.001$ versus 5×10^{10} gc/mouse (one-way ANOVA test).

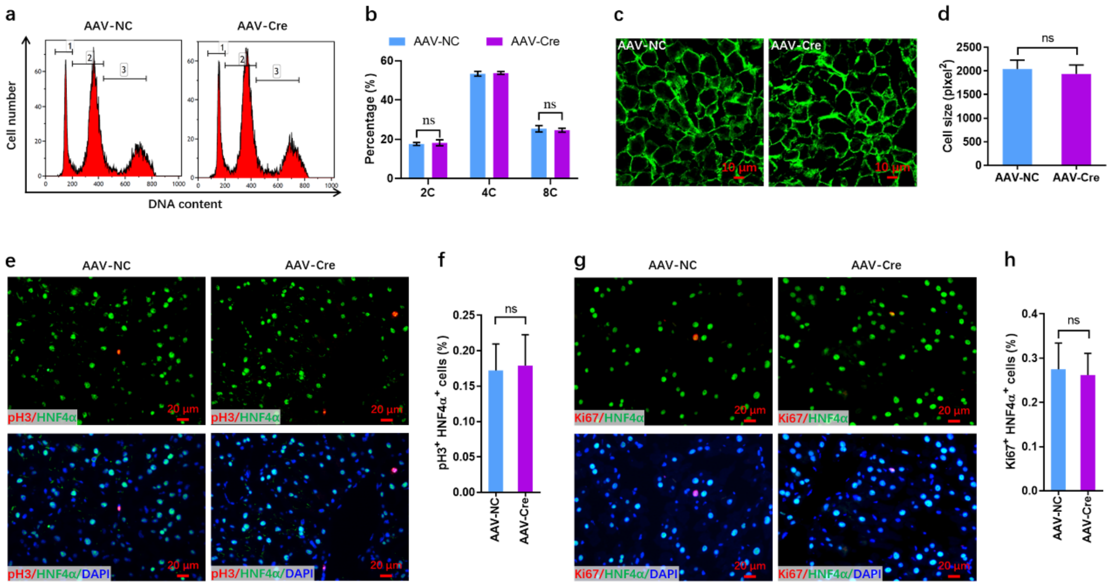

Supplementary Figure 12. Effects of the AAV-Cre-mediated deletion of FoxO3 on hepatocyte ploidy and proliferation at 0 dpH. (a and b) Representative images (a) and quantification (b) of the ploidy contribution of primary hepatocytes isolated from control and AAV-Cre mice at 0 dpH are shown. Peaks 1, 2 and 3 depicted, respectively, 2C, 4C and 8C cells. Data are presented as the mean ± SEM (*n*=3 mice per group), ns denotes no significant difference. (c and d) Representative images of β-catenin staining (c) and quantification of hepatocyte size (d) in livers from control and AAV-Cre mice at 0 dpH. Data are presented as the mean ± SEM (*n*=5 mice per group), ns denotes no significant difference. (e and f) Representative images (e) and quantification (f) of pH3$^+$ HNF4α$^+$ cells in control and AAV-Cre mice at 0 dpH. Data are presented as the mean ± SEM (*n*=5 mice per group), ns denotes no significant difference. (g and h) Representative images (g) and quantification (h) of Ki67$^+$ HNF4α$^+$ cells in control and AAV-Cre mice at 0 dpH. Data are presented as the mean ± SEM (*n*=5 mice per group), ns denotes no significant difference.

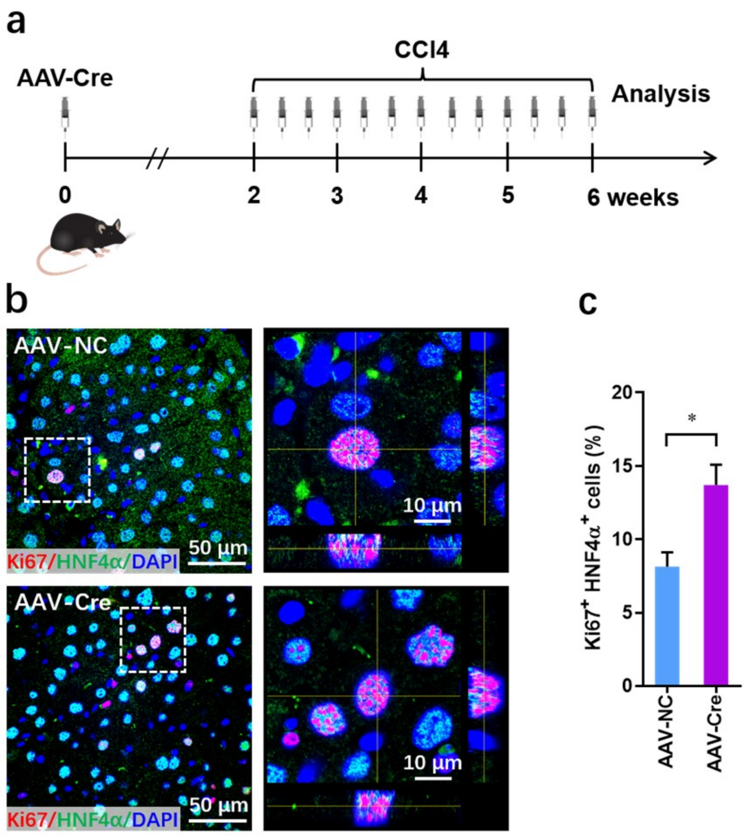

Supplementary Figure 13. Effects of FoxO3 deficiency specifically in adult liver on hepatocyte proliferation upon CCl4-induced injury. (a) Schematic of AAV-Cre virus injection and liver injury model induction with CCl4 in adult FoxO3$^{fl/fl}$ mice. (b and c) Representative images (b) and quantification (c) of Ki67^{+} HNF4α^{+} hepatocytes in livers upon CCl4-induced injury. Data are presented as the mean ± SEM (*n*=5 mice). **p*<0.05 versus controls (Student's *t*-test).

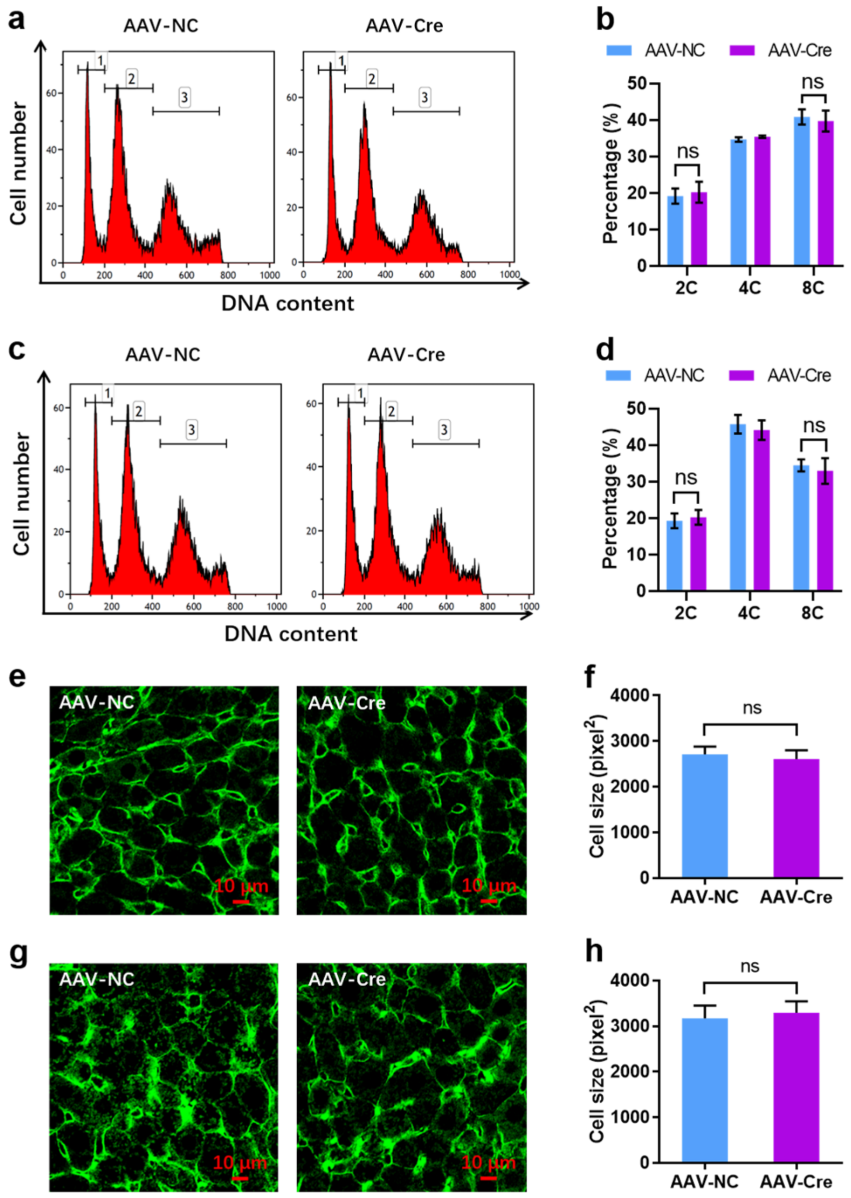

Supplementary Figure 14. Effects of the AAV-Cre-mediated deletion of FoxO3 on the ploidy and size of hepatocytes during liver regeneration. (a and b) Representative images (a) and quantification (b) of the ploidy contribution of primary hepatocytes isolated from control and AAV-Cre mice at 2 dpH are shown. Peaks 1, 2 and 3 depicted, respectively, 2C, 4C and 8C cells. Data are presented as the mean ± SEM (*n*=3 mice per group), ns denotes no significant difference. (c and d) Representative images (c) and quantification (d) of the ploidy contribution of primary hepatocytes isolated from control and AAV-Cre mice at 4 dpH are shown. Peaks 1, 2 and 3 depicted, respectively, 2C, 4C and 8C cells. Data are presented as the mean ± SEM (*n*=3 mice per group), ns denotes no significant difference. (e and f) Representative images of β-catenin staining (e) and quantification of hepatocyte size (f) in livers from control and AAV-Cre mice at 2 dpH. Data are presented as the mean ± SEM (*n*=5 mice per group), ns denotes no significant difference. (g and h) Representative images of β-catenin staining (g) and quantification of hepatocyte size (h) in livers from control and AAV-Cre mice at 4 dpH. Data are presented as the mean ± SEM (*n*=5 mice per group), ns denotes no significant difference.

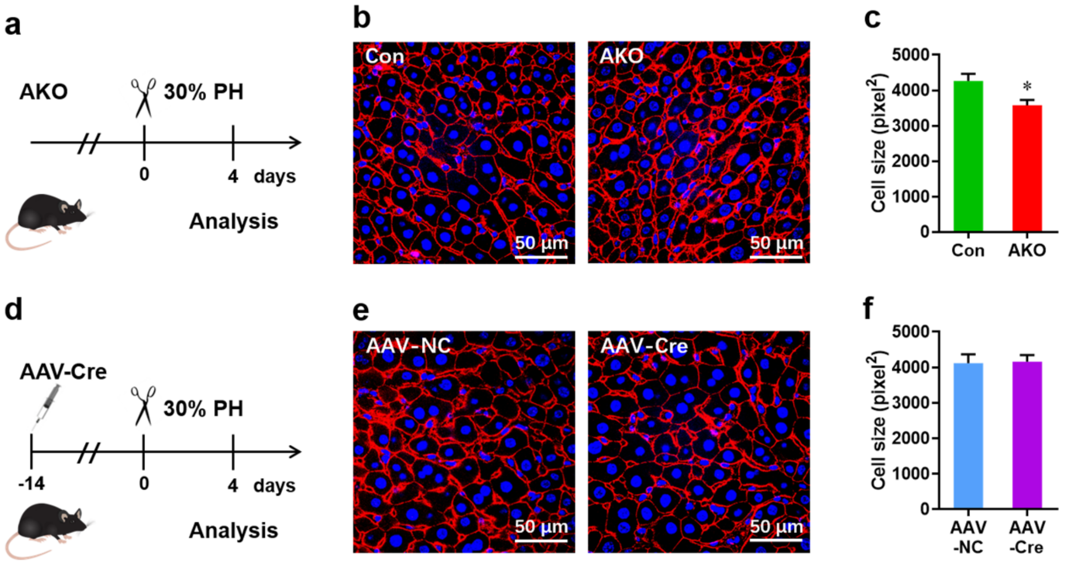

Supplementary Figure 15. Effects of FoxO3 deficiency on hepatocyte sizes at 4 dpH upon 30% PH injury. (a) Schematic of 30% PH injury model induction and liver tissue collection in adult AKO mice. (b and c) Representative images of β-catenin staining (b) and quantification of hepatocyte size (c) in livers from control and AKO mice at 4 dpH. Data are presented as the mean ± SEM (n=5 mice per group), *p<0.05 versus control. (d) Schematic of 30% PH injury model induction, AAV-Cre virus injection and liver tissue collection in adult FoxO3$^{fl/fl}$ mice. (e and f) Representative images of β-catenin staining (e) and quantification of hepatocyte size (f) in livers from control and AAV-Cre mice at 4 dpH. Data are presented as the mean ± SEM (n=5 mice per group), *p<0.05 (Student's t-test).

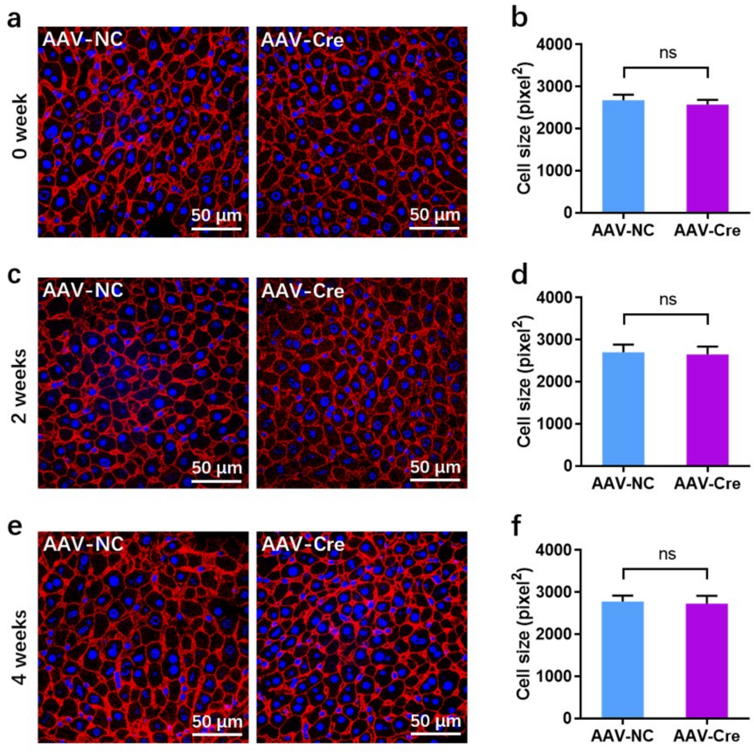

Supplementary Figure 16. Effects of FoxO3 deficiency on hepatocyte sizes during homeostasis. Adult FoxO3$^{fl/fl}$ mice were injected with AAV-Cre and control viruses, followed by hepatocyte size assay at indicated time points. (a and b) Representative images of β-catenin staining (a) and quantification of hepatocyte size (b) in livers from control and FoxO3-deficient mice at 0 week post-Injection (wpI). (c and d) Representative images of β-catenin staining (c) and quantification of hepatocyte size (d) in livers from control and FoxO3-deficient mice at 2 wpI. (e and f) Representative images of β-catenin staining (e) and quantification of hepatocyte size (f) in livers from control and FoxO3-deficient mice at 4 wpI. Data are presented as the mean ± SEM (*n*=5 mice per group).

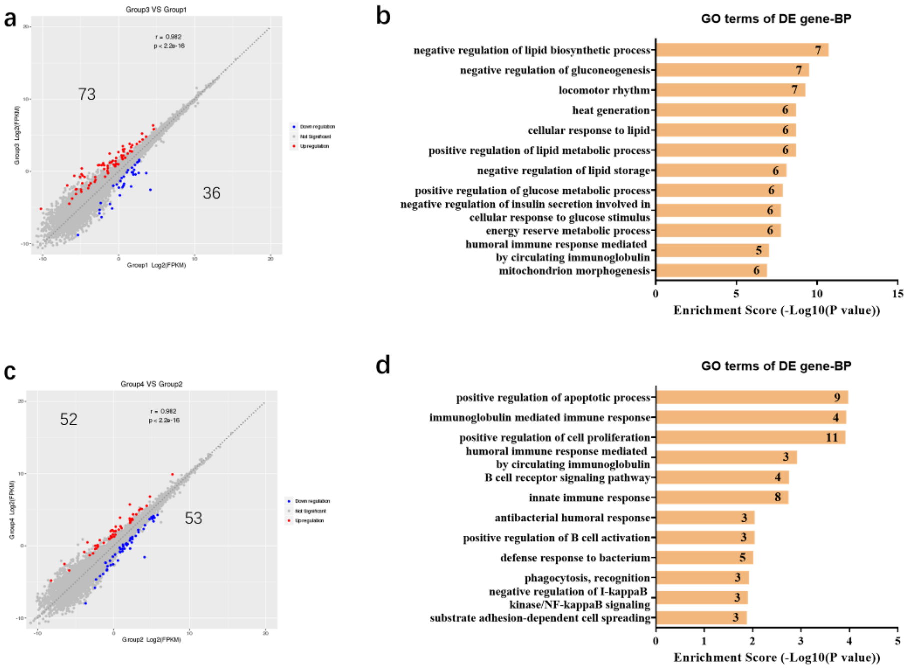

Supplementary Figure 17. *FoxO3* knockout leads to altered expression of target genes relative to proliferation and apoptosis in livers at 4dpH. (a) Overall changes of genes in *FoxO3*-knockout mice before PH compared with control mice. (b) GO analysis of differentially expressed genes in *FoxO3*-knockout mice before PH compared with control mice. (c) Overall changes of genes in FoxO3-knockout mice at 4dpH compared with control mice. (d) GO analysis of differentially expressed genes in FoxO3-knockout mice at 4dpH compared with control mice.

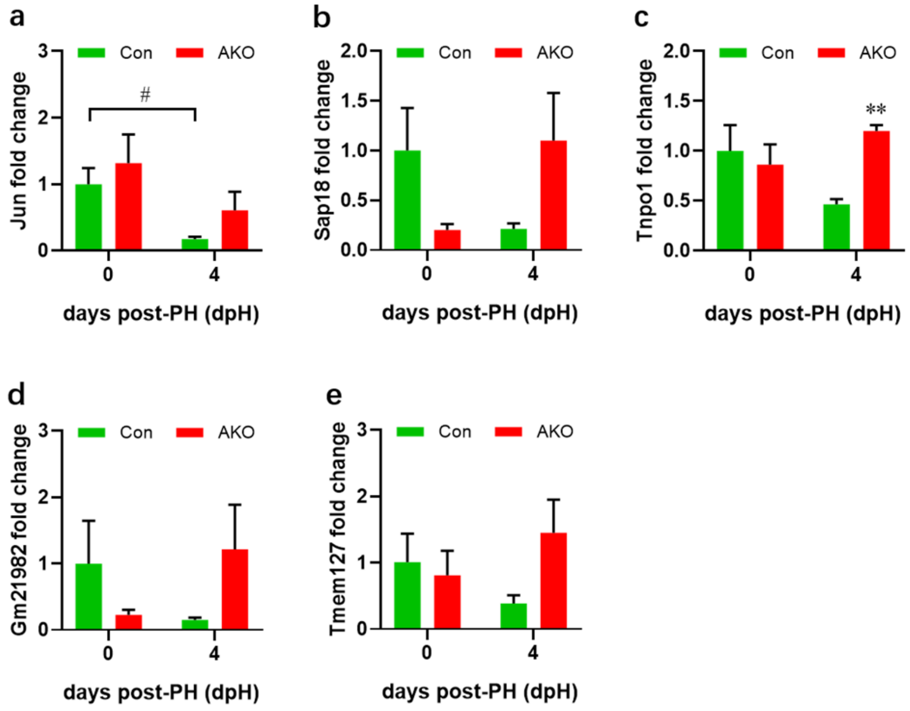

Supplementary Figure 18. Effects of PH on the expression of the overlapping genes (relative to Figure 7A). qPCR validation of *Jun* (a), *Sap18* (b), *Tnpo1* (c), *Gm21982* (d), and *Tmem127* (e) liver expression in AKO mice before and after PH compared with control mice (*n*=3 mice per group). Data are presented as the mean ± SEM. $^{**}p<0.01$ versus controls, $^{\#}p<0.05$ (two-way ANOVA test).

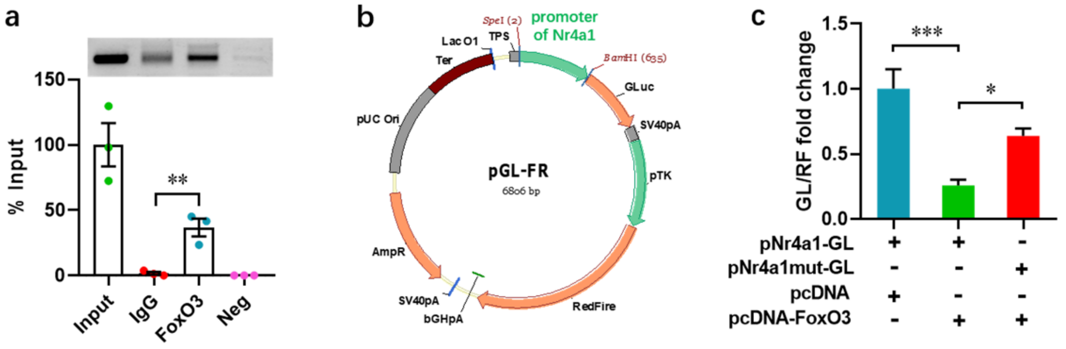

Supplementary Figure 19. Interaction between FoxO3 and the *Nr4a1* promoter. (a) Representative image of ChIP-PCR assay using liver tissues (up panel) and relative quantification of the *in vivo* binding ability of FoxO3 with the *Nr4a1* promoter (n=3 mice). DNA signals for input are set to 100%. Neg, negative control without DNA template. (b) Schematic of the luciferase reporter plasmid used to evaluate the interaction of FoxO3 with the *Nr4a1* promoter. (c) Quantification of the relative luciferase activity of NCTC1469 cells with and without *FoxO3* overexpression (n=4 per group). All data are presented as the mean ± SEM. *p<0.05, **p<0.01, ***p<0.001 (one-way ANOVA test).

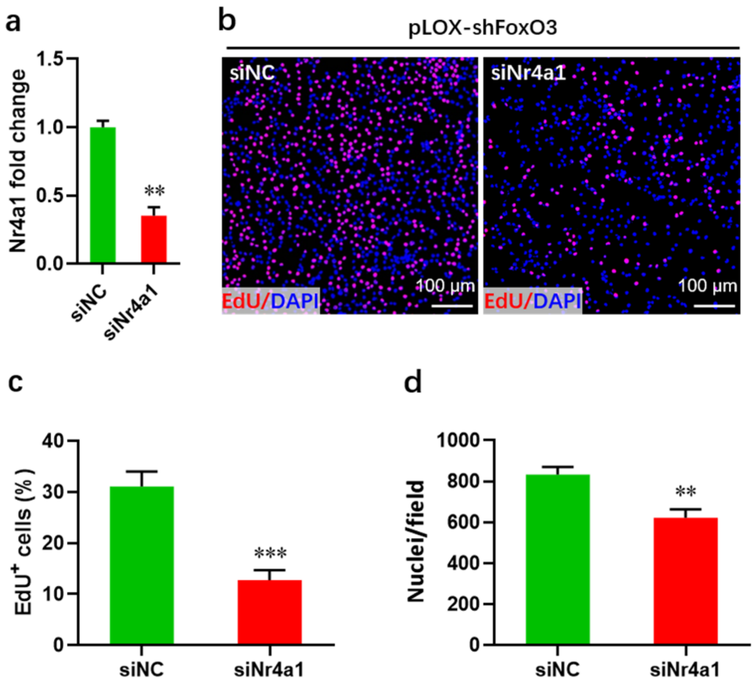

Supplementary Figure 20. *Nr4a1* silencing suppresses the proliferation of FoxO3-deficient NCTC1469 cells. (a) qPCR validation of *Nr4a1* knockdown in NCTC1469 cells (*n*=3 per group). (b-d) Representative images (b) and quantification of EdU$^+$ cells (c) and cell density (d) in *FoxO3*-deficient NCTC1469 cells transfected with siNr4a1 and siNC (*n*= 6 per group). All data are presented as the mean ± SEM. **p<0.01 versus controls (Student's t-test).

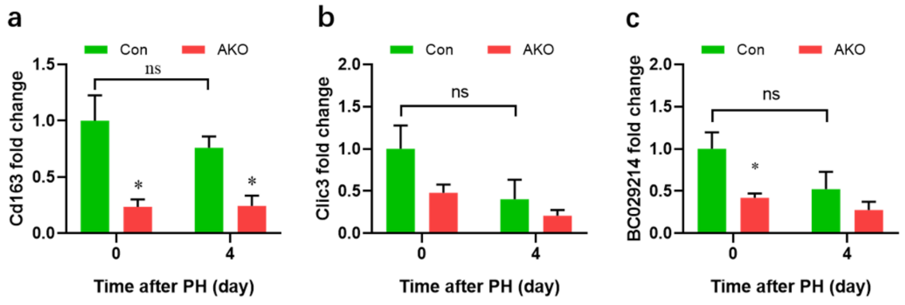

Supplementary Figure 21. Effects of PH on the expression of the overlapping genes (relative to Figure 8A). qPCR validation of *Cd163* (a), *Clic3* (b), and *BC029214* (c) liver expression in AKO mice before and after PH compared with control mice (n=3 mice per group). Data are presented as the mean ± SEM. *p<0.05 versus controls, ns denotes no significant difference (two-way ANOVA test).

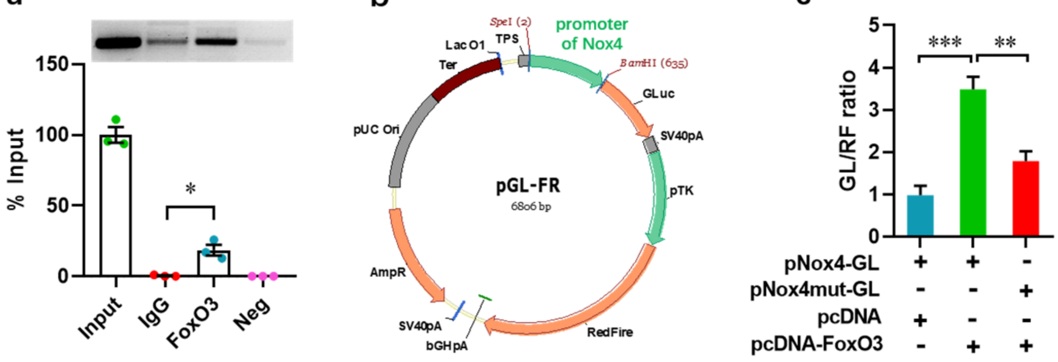

Supplementary Figure 22. Interaction between FoxO3 and the *Nox4* promoter. (a) Representative image of ChIP-PCR assay using liver tissues (up panel) and relative quantification of the *in vivo* binding ability of FoxO3 with the *Nox4* promoter (n=3 mice). DNA signals for input are set to 100%. Neg, negative control without DNA template. (b) Schematic of the luciferase reporter plasmid used to evaluate the interaction of FoxO3 with the *Nox4* promoter. (c) Quantification of the relative luciferase activity of NCTC1469 cells with and without *FoxO3* overexpression (n=4 per group). All data are presented as the mean ± SEM. *p<0.05, **p<0.001, ***p<0.001 (one-way ANOVA test).

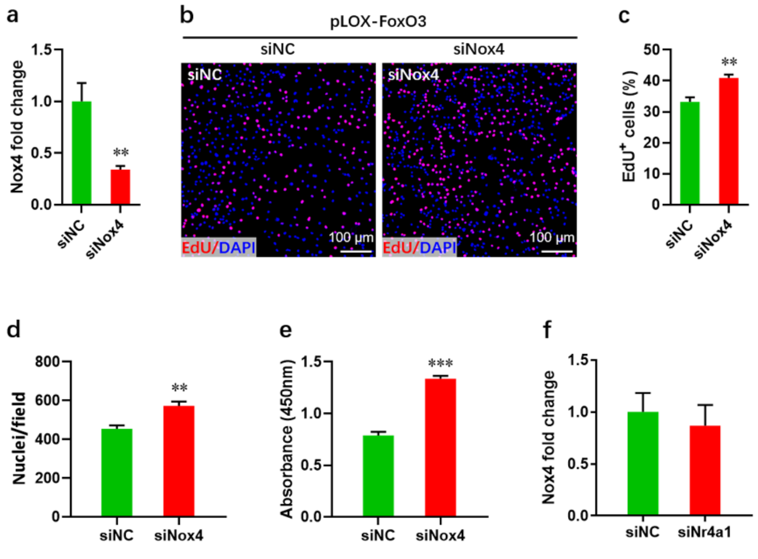

Supplementary Figure 23. *Nox4* silencing promotes the proliferation of FoxO3-overexpresing NCTC1469 cells. (a) qPCR validation of *Nox4* knockdown in NCTC1469 cells (*n*=5 per group). (b-d) Representative images (b) and quantification of EdU^{+} cells (c) and total cells (d) in *FoxO3*-overexpressing NCTC1469 cells transfected with siNox4 and siNC (*n*=6 per group). (e) Proliferation was examined by cell counting assay in FoxO3-overexpressing NCTC1469 cells transfected with siNox4 or siNC (*n*=10 per group). (f) Effects of Nr4a1 knockdown on the expression of Nox4 in NCTC1469 cells (*n*=4 per group). All data are presented as the mean ± SEM, $**p<0.01$, $***p<0.001$ versus controls (Student's *t*-test).

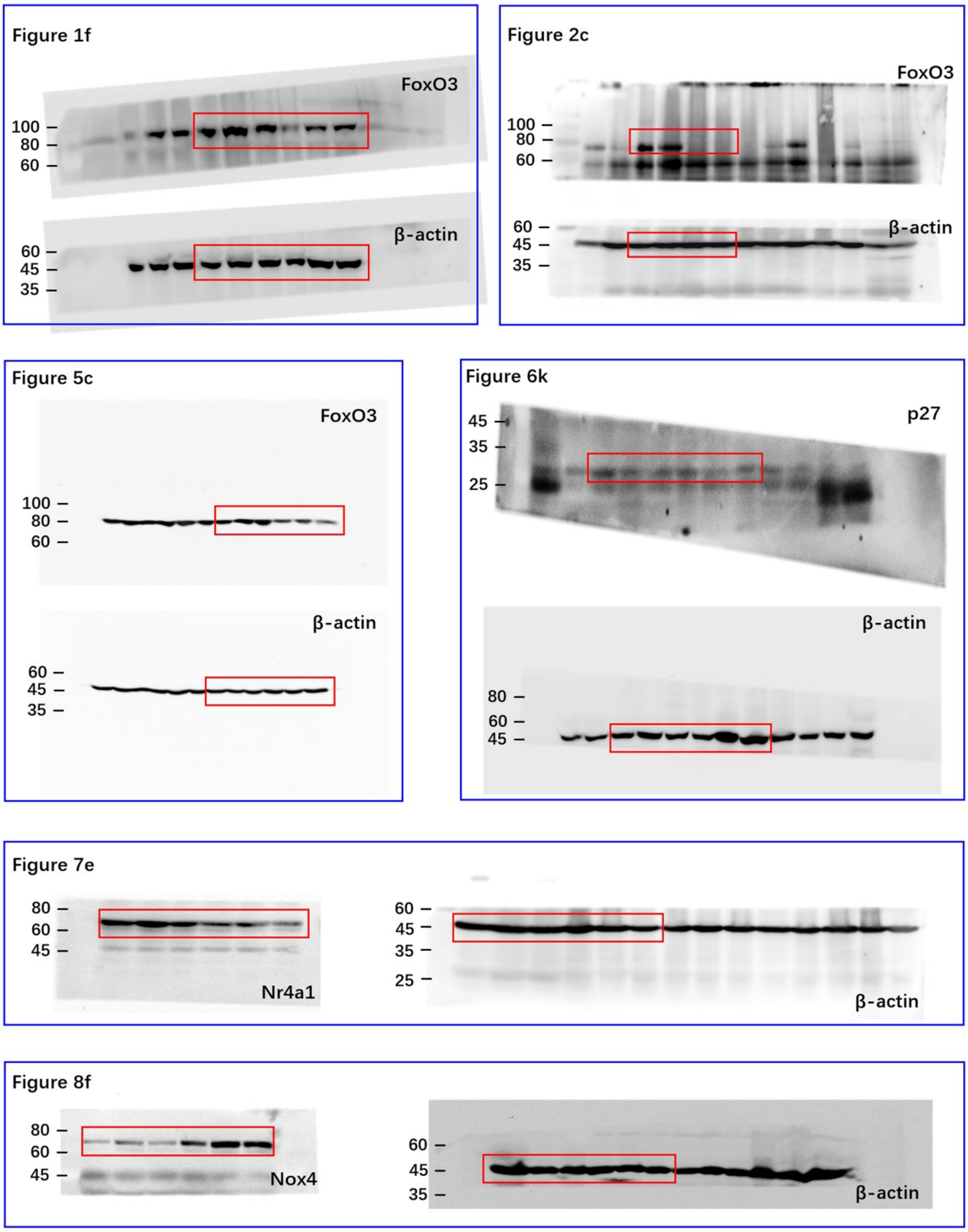

Supplementary Figure 24. Uncropped blots relate to figures in this study. Red square frames indicate the representative bands used in the main text.

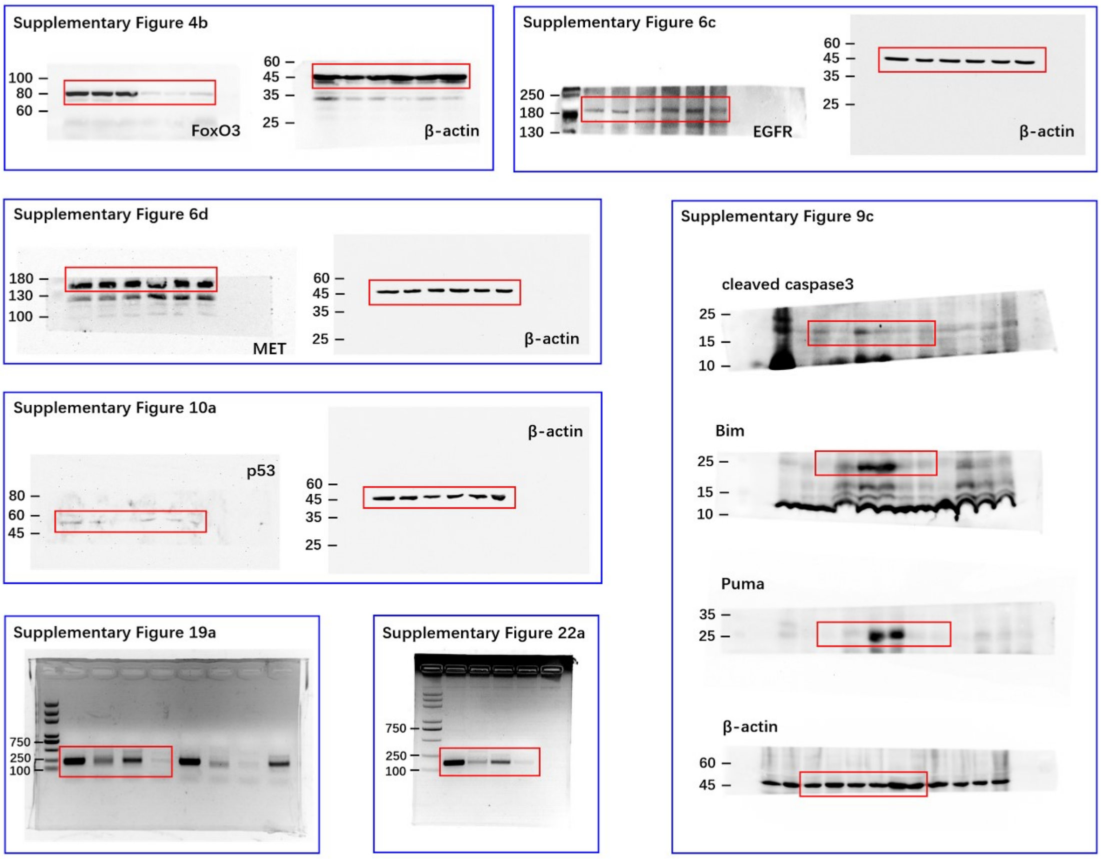

Supplementary Figure 25. Uncropped blots relate to supplementary figures in this study. Red square frames indicate the representative bands used in the main text.

Supplementary Table 1. FoxO3 binding sites in the promoter regions of mice *Nr4a1*.

Matrix ID	Name	Score	Relative score	Start	End	Predicted sequence
MA0157.1	FOXO3	10.2054	0.934945	197	204	ggaaaaca
MA0157.1	FOXO3	9.76145	0.920032	1686	1693	tggaaaca
MA0157.1	FOXO3	9.37963	0.907206	849	856	tgtaaata
MA0157.1	FOXO3	8.83822	0.889018	560	567	agtaaaca
MA0157.1	FOXO3	8.30279	0.871033	625	632	tgtaaaga
MA0157.1	FOXO3	6.95915	0.825897	1349	1356	tgaaaaaa
MA0157.1	FOXO3	6.85145	0.822279	639	646	tgaaacca

The promoters (-2,000 to -1, upstream of TSS) of target gene were predicted by JASPAR 2018 online software. The binding site with highest score (red) was further analyzed by luciferase report gene system.

Supplementary Table 2. FoxO3 binding sites in the promoter regions of mice *Nox4*.

Matrix ID	Name	Score	Relative score	Start	End	Predicted sequence
MA0157.1	FOXO3	10.7984	0.954864	1467	1474	tgaaaaca
MA0157.1	FOXO3	10.7984	0.954864	316	323	tgaaaaca
MA0157.1	FOXO3	9.76145	0.920032	135	142	tggaaaca
MA0157.1	FOXO3	8.88869	0.890714	350	357	tcaaaaca
MA0157.1	FOXO3	8.30279	0.871033	1581	1588	tgtagaca
MA0157.1	FOXO3	8.03598	0.86207	1428	1435	tgaaaata
MA0157.1	FOXO3	7.95283	0.859277	1275	1282	gttaaaca
MA0157.1	FOXO3	7.70982	0.851113	274	281	ggtagaca
MA0157.1	FOXO3	7.70982	0.851113	822	829	ggtagaca
MA0157.1	FOXO3	7.70982	0.851113	1197	1204	ggtaaaaa
MA0157.1	FOXO3	7.70982	0.851113	1645	1652	ggtaaaga
MA0157.1	FOXO3	7.46993	0.843055	416	423	tctacaca
MA0157.1	FOXO3	6.95915	0.825897	713	720	tgaaaacc
MA0157.1	FOXO3	6.95915	0.825897	1084	1091	tgaaaaaa
MA0157.1	FOXO3	6.45764	0.80905	248	255	aggaaaca

The promoters (-2,000 to -1, upstream of TSS) of target gene were predicted by JASPAR 2018 online software. The binding site with highest score (red) was further analyzed by luciferase report gene system.

Supplementary Table 3. siRNAs used in this study for gene silencing in mice.

**Target gene**	**siRNA (5'-3')**
Nr4a1	GGACAGAGCAGTTGCCTAA
Nox4	CCATTTGCATCGATACTAA
Puma	GAGACAAGAAGAGCAGCAT
Bim	GGAGGGTGTTTGCAAATG
p27	GCAAGTGGAATTTCGACTT
Negative control (NC)	GTCCAGATTGTCCGCAACTA

Supplementary Table 4. List of antibodies with detailed information used in this study.

Epitope	Species	Supplier	Catalogue number	Clone	Dilution	Antigen Retrieval
**Immunofluorescence**						
FoxO3	Rabbit	CST	#12829	Monoclonal	1:500	Cit
HNF4α	Rabbit	Abcam	ab201460	Monoclonal	1:500	Cit
pH3	Mouse	CST	#9706	Monoclonal	1:800	Cit
Ki67	Rabbit	Abcam	ab15580	Polyclonal	1:1000	Cit
PCNA	Mouse	Sigma	SAB4200708	Monoclonal	1:1000	Cit
E-cadherin	Mouse	R&D	AF748	Polyclonal	10μg/mL	Cit
Glutamine synthetase	Rabbit	Abcam	ab49873	Polyclonal	1:1000	Cit
**Immunohistochemistry**						
Cleaved caspase-3	Rabbit	CST	#9661	Polyclonal	1:200	Cit
Ki67	Rabbit	Abcam	ab15580	Polyclonal	1:250	Cit
**WB**						
FoxO3	Rabbit	CST	#12829	Monoclonal	1:1000	
p27	Mouse	CST	#3698	Monoclonal	1:1000	
Bim	Rabbit	CST	#2933	Monoclonal	1:1000	
Puma	Rabbit	CST	#24633	Monoclonal	1:1000	
Cleaved caspase-3	Rabbit	CST	#9661	Polyclonal	1:1000	
p53	Rabbit	Abcam	ab131442	Polyclonal	1:250	
Nr4a1	Rabbit	Proteintech	25851-1-AP	Polyclonal	1:500	
Nox4	Mouse	Santa Cruz	sc-518092	Monoclonal	1:500	
EGFR	Rabbit	CST	#4267	Monoclonal	1:1000	
MET	Rabbit	Abcam	ab254252	Monoclonal	1:1000	
β-actin	Mouse	Proteintech	66009-1-Ig	Monoclonal	1:2000	
**ChIP**						
FoxO3	Rabbit	CST	#2497	Monoclonal	1:100	
Normal Rabbit IgG	Rabbit	CST	#2729	Polyclonal	1:100	

Cit means Citrate Antigen Retrieval Solution pH 6.0 (Beyotime Biotechnology, P0081).

Supplementary Table 5. Primer sequences for real-time PCR analysis in mice.

Gene	Primer sequence (5'-3')	
	Forward primer	Reverse primer
FoxO3	TACGAGTGGATGGTGCGCTGT	TCATTCTGAACGCGCATGAAGC
Ccnd1	GAAGGAGACCATTCCCTTGA	GTTCACCAGAAGCAGTTCCA
Ccne1	CAAACTCAACGTGCAAGCCTC	GCCCAGCTCAGTACAGGCAG
P21	ATCACCAGGATTGGACATGG	CGGTGTCAGAGTCTAGGGGA
P27	TCAAACGTGAGAGTGTCTAACG	CCGGGCCGAAGAGATTTCTG
P57	GCAGGACGAGAATCAAGAGCA	GCTTGGCGAAGAAGTCGTT
Nr4a1	CTTCCTGCTCAGTTCCTCGC	ATGGTAGGCTTGCCGAACTC
Nox4	TGTTGGGCCTAGGATTGTGT	CAGGACTGTCCGGCACATAG
Jun	GCACATCACCACTACACCGA	GGGAAGCGTGTTCTGGCTAT
Sap18	TGTTCAATCAGCCCAGGTGT	GGCTTAAAATGCCCTCTACTGC
Tnpo1	TTCTCTTTGTTCCGCAGCCA	TGCTCGTCAGGTTTCCACTC
Gm21982	AAAGAGCGGGAACTGAGTCG	CCTCTGACTACGCACATGCT
Tmem127	TTGGGGCCTGGGATCGTTTG	CAGAGCCTGTCGGTCAGCAG
Cd163	TGCTGTCACTAACGCTCCTG	TCATTCATGCTCCAGCCGTT
Clic3	CCACCATGGCTGAAACCACC	ATCCAATGCCCTTCGAGTGT
BC029214	CCAAGAAGAACACCCAGCCA	GCTTTGCTGTCCTCAGGTCT
Smad7	ACAGAGGATCTTGTCCCCGA	CGTCTCAGGCAGCTCTCTC
Serpine1	AGCTTTGTGAAGGAGGACCG	AAGGTGCCTTGTGATTGGCT
Col1a1	TTCTCCTGGCAAAGACGGAC	CGGCCACCATCTTGAGACTT
Col1a2	CCCAGAGTGGAACAGCGATT	ATGAGTTCTTCGCTGGGGTG
Gapdh	TGTGTCCGTCGTGGATCTGA	TTGCTGTTGAAGTCGCAGGAG
